# Visible light-induced direct α C–H functionalization of alcohols

**DOI:** 10.1038/s41467-019-08413-9

**Published:** 2019-01-28

**Authors:** Linbin Niu, Jiamei Liu, Xing-An Liang, Shengchun Wang, Aiwen Lei

**Affiliations:** College of Chemistry and Molecular Sciences, the Institute for Advanced Studies (IAS), Wuhan University, Wuhan, Hubei 430072 China

## Abstract

Considering the synthetic value of introducing active alcoholic hydroxyl group, developing C–H functionalization of alcohols is of significance. Herein, we present a photochemical method that under visible light irradiation, selectfluor can effectively promote the oxidative cross-coupling between alcohols and heteroarenes without the external photocatalysis, achieving the selective α sp^3^ C–H arylation of alcohol, even in the presence of ether. The N-F activation of selectfluor under blue LEDs irradiation is evidenced by electron paramagnetic resonance (EPR) study, which is the key process for the oxidative activation of α sp^3^ C–H alcohols. The observed reactivity may have significant implications for chemical transformations.

## Abstract (web summary)

Alcohols are very useful building blocks in organic synthesis, however C–H functionaliztion in presence of free –OH groups is highly challenging. Here, the authors report a selective visible light-promoted α-functionalization of free alcohols with heteroarenes mediated by Selectofluor.

## Introduction

**Fig. 1:**
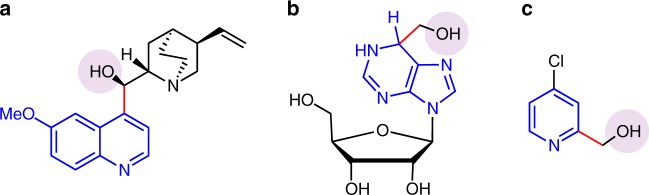
Important molecules containing alcoholic hydroxyl groups. **a** Anti-malarial natural product quinine. **b** Inhibitor of adenosine deaminase. **c** Inhibitor of gastric acid secretion

Alcohols as one of the most common raw chemical materials, are indispensable to organic chemistry and chemical engineering. The presence of hydroxyl group enables them to play diverse roles such as good solvents, competent nucleophiles^1–5^, suitable directing group^6–8^, and frequently used proton source for a long time^9–11^. The strategies, sp^3^ C–H functionalization of alcohols including α sp^3^ C–H functionalization and remote sp^3^ C–H functionalization^12–15^, which can transform alcohols into value-added chemicals, are significant for the organic synthesis and bio-pharmaceuticals (Fig. 1)^16,17^.

**Fig. 2:**
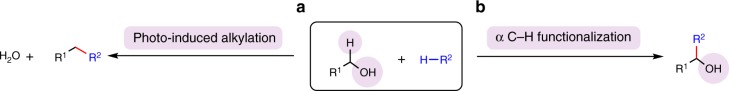
The transformation of alcohols. **a** Photo-induced alkylation of heteroarenes by using alcohols as alkylating reagent. **b** α sp^3^ C–H functionalization of alcohols

During the past decade, photoredox catalysis featured by the conversion of light energy into chemical energy and single electron transfer (SET) events, has facilitated the discovery of numerous elegant and challenging chemical transformations^18–26^. Particularly, impressive studies on employing the alcohols as alkylating reagent with the loss of alcoholic hydroxyl groups—achieved photochemical alkylation of electron-deficient heteroarenes (Fig. 2a)^27–30^. Considering the multiple functionality of alcoholic hydroxyl group in bioactive molecules and the frequency of its use as synthetic handles, selective functionalization of α sp^3^ C–H of alcohols via a photochemical process is undeniably attractive for its applications in synthetic organic chemistry (Fig. 2b)^31^.

To enable selective functionalization of α sp^3^ C–H of alcohols to introduce the alcoholic hydroxyl group, enormous efforts have been made by chemists. For example, remarkable works on the cross-coupling between alcohols with unsaturated bonds such as alkenes, allenes and alkynes have been extensively reported^32–40^, providing effective routes for α sp^3^ C–H activation and functionalization of alcohols. Furthermore, oxidation-induced C–H functionalization as a powerful tool^41–48^, is successfully applied in the α sp^3^ C–H functionalization of alcohols^49–58^. Direct oxidative α sp^3^ C–H arylation by C–H/C–H cross-coupling to acquire the modified alcohols is undoubtedly the most step- and atom-economical method. It is worth noting that peroxide-mediated oxidative arylation of alcohols with different heterocycles predominates this topic^59,60^. Herein, we describe an oxidative α sp^3^ C–H arylation of alcohols with heterocycles promoted by selectfluor under visible light irradiation, which is selective for the α sp^3^ C–H of alcohols, even in the presence of ethers. The N–F activation of selectfluor by blue light emitting diodes (LEDs) irradiation is evidenced by EPR studies. The observed reactivity may have important implications for sp^3^ C–H functionalization.

## Results

### Exploration of reaction pathways

**Fig. 3:**
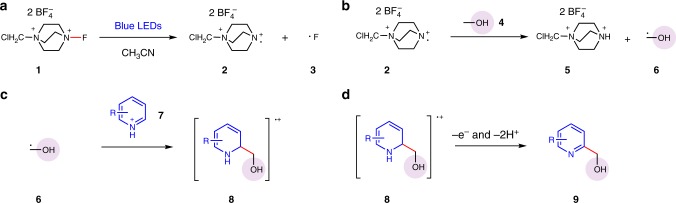
The designed reaction pathways. **a** The N–F activation of selectfluor under blue LEDs irradiation. **b** The hydrogen-atom transfer (HAT) between generated N radical cation with alcohols to yield hydroxyalkyl radical. **c** The nucleophilic addition of hydroxyalkyl radical to electron-deficient heteroarenes. **d** The oxidative aromatization of the radical adducts to the final product

Selectfluor **1** is well-known as a powerful fluorination reagent and oxidant, frequently combined with a metal catalyst or photocatalyst in the organic synthesis^61–68^. The N–F breakage of selectfluor resorts to the immigration of external electron from a reductant. We questioned whether the visible light irradiation could induce the N–F activation of selectfluor to directly yield the corresponding N radical cation **2** and F radical **3** (Fig. 3a). The generated N radical cation **2** is responsible for the abstraction of α sp^3^ C–H of alcohol **4** to the hydroxyalkyl radical **6** (Fig. 3b). Afterward, the electron-deficient heteroarenes **7** protonated by acid can capture the relatively nucleophilic radical and deliver the corresponding radical adducts **8** (Fig. 3c). The oxidation and deprotonation of this radical adduct **8** by another selectfluor would then afford the α-arylated product **9** (Fig. 3d). The key difference between this oxidative α sp^3^ C–H arylation of alcohols and those reported photochemical alkylation of heteroarenes^28–30^, is the oxidation condition. Under the designed oxidation condition, the spin center shift process of the intermediate **8** can be avoided and the alcoholic hydroxyl group is unaffected, achieving the oxidative α sp^3^ C–H arylation of alcohols with heteroarenes.

**Fig. 4:**
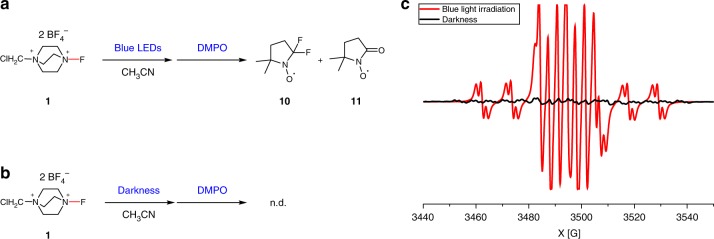
The electron paramagnetic resonance (EPR) experiments. **a** The EPR experiment of selectfluor under blue light irradiation. **b** The EPR experiment of selectfluor under darkness. **c** The EPR spectra of selectfluor under blue light irradiation and darkness

This assumption that the N–F activation of selectfluor could be achieved by blue light irradiation was evidenced by EPR experiments (see Supplementary Methods). Two kinds of radical signals were observed, when selectfluor in acetonitrile was irradiated by blue LEDs and 5,5-dimethyl-1-pyrroline *N*-oxide (DMPO) was employed as a radical scavenger. Fitting the EPR spectra on the basis of electron spin resonance parameters of spin adducts^69^, one of the radical **10** was confirmed as the radical adduct between two fluorine radical and DMPO, while the other one **11** was resulted from the oxidation of DMPO, whose ratio is 3:8 (Fig. 4a, c). In contrast, we did not detect the radical adduct between two fluorine radical and DMPO under the darkness (Fig. 4b, c).

### Investigation of reaction conditions

With the mechanistic evidence in hand, we started our investigations with isoquinoline **12** and ethanol as the model substrates. We identified that using selectfluor as a visible light-activated oxidant irradiated by blue LEDs, in the presence of 1.5 equiv trifluoroacetic acid (TFA), the desired oxidative α sp^3^ C–H arylation product can be afforded in 87% yield (Table 1, entry 1). It is noteworthy that upon treatment of this reaction with green light irradiation or darkness, no product was detected (Table 1, entries 2 and 3). Even if the reaction system was heated to 80 °C under darkness, the conversion was still not promoted (Table 1, entry 4). These results might reveal that only the shorter wavelength visible light, which possesses the higher energy could achieve the N–F activation of selectfluor and then promote the oxidative α sp^3^ C–H arylation of alcohol, while the longer wavelength visible light and heating failed. In addition, the contemporary fluorination reagents N-fluorobenzenesulfonimide was also examined under the same condition with blue light irradiation. However, a poor reactivity was observed (Table 1, entry 5), probably resulted from the higher bond dissociation energy of N–F compared with selectfluor^70^. Although the common-used oxidants such as *t*-butylhydroperoxide (TBHP), potassium persulfate (K_2_S_2_O_8_), and (diacetoxyiodo)benzene (PhI(OAc)_2_) show efficient capacity for oxidative activation of sp^3^ C–H, the reaction still could not be promoted by utilizing these oxidants at 80 °C (Table 1, entries 6–8), implying the uniqueness of selectfluor under blue light irradiation for the oxidative α sp^3^ C–H arylation of alcohols.

**Table 1:** Investigation of the reaction conditions.*


Entry	Oxidant	Light source	T (°C)	Yield (%)^†^
1	Selectfluor	Blue LEDs	25	87
2	Selectfluor	Green LEDs	25	N.D.
3	Selectfluor	Darkness	25	N.D
4	Selectfluor	Darkness	80	Trace
5	NFSI	Blue LEDs	25	Trace
6	TBHP	Darkness	80	N.D.
7	K_2_S_2_O_8_	Darkness	80	N.D.
8	PhI(OAc)_2_	Darkness	80	N.D.

^*^Conditions: **12** (0.3 mmol), ethanol (1.5 mL), selectfluor (0.6 mmol), TFA (0.45 mmol), in CH_3_CN (2.0 mL) under a nitrogen atmosphere, irradiated with 3 W blue LEDs at 25 °C for 24 h; *N.D.* not detected

^†^Isolated yield

### Substrate scope

**Fig. 5:**
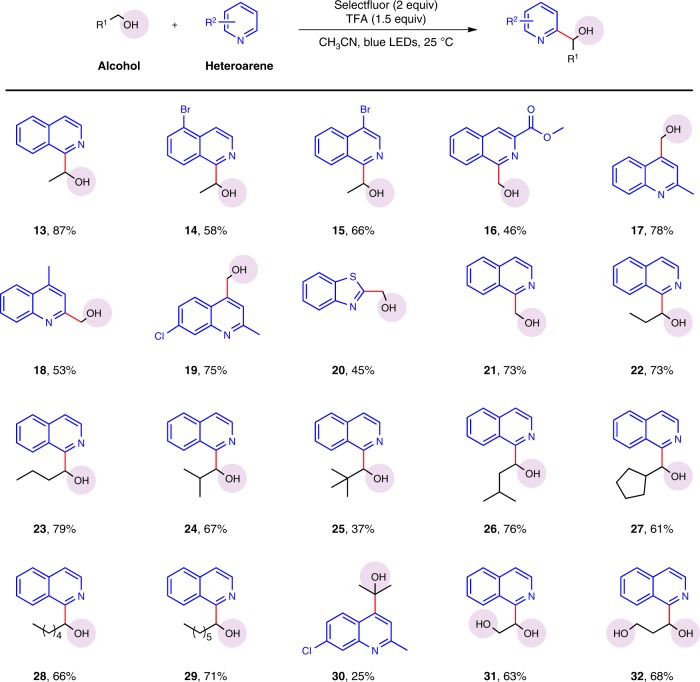
Substrate scope for the α sp^3^ C–H arylation of alcohols with heteroarenes. Reaction conditions: heteroarene (0.3 mmol), alcohol (see Supplementary Methods for details), selectfluor (0.6 mmol), TFA (0.45 mmol), in CH_3_CN (2.0 mL) (additional 0.75 mL DCE was added for **25**) under a nitrogen atmosphere, irradiated with 3 W blue LEDs at 25 °C for 24 h

With the optimized conditions established, we hoped this method could be applied to other noble heteroaromatics (Fig. 5). Isoquinolines with halides and esters substituents are competent functionalization partners, successfully delivering the desired oxidative α sp^3^ C–H arylation products (**14**–**16**). It was found that the quinoline derivatives such as methyl and halides substituted quinolines performed good reactivities (**17**–**19**). Importantly, the addition of methanol to benzothiazole could be smoothly proceeded under the standard condition (**20**). Unfortunately, the reactivity of pyridine and pyrazine was poor under the same catalytic system. Subsequently, a variability of alcohols were examined in details. Methanol, *n*-propanol and *n*-butyl alcohol were effectively oxidized to corresponding nucleophilic radicals and reacted with isoquinoline in good to high yields under the photochemical condition (**21**–**23**). When the ethanols containing isopropyl, *tert*-butyl, isobutyl and cyclopentyl were tested, we still isolated the oxidative arylation products with moderate yields, in spite of the steric hindrance proximal to the α sp^3^ C–H of alcohols (**24**–**27**). It is worth noting that long-chain alkyl alcohols like *n*-hexanol and *n*-heptanol are also suitable for this protocol (**28** and **29**). Isopropanol could also be tolerated, even though a low yield was obtained (**30**). Notably, dioles were successfully tolerated, delivering the modified monoarylation dioles (**31** and **32**).

**Fig. 6:**
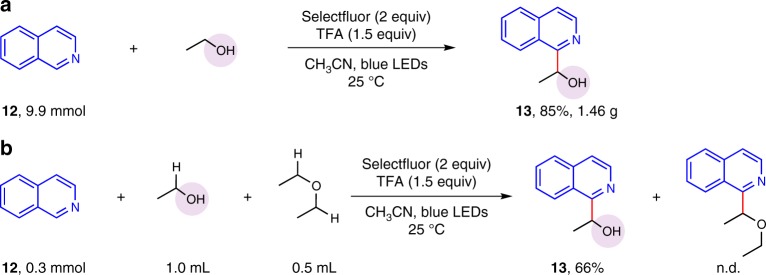
Investigation and application of this protocol. **a** Gram-scale synthesis experiment. **b** Intermolecular competition experiment

Additionally, the gram-scale synthesis experiment was carried out (see Supplementary Methods). A comparable yield 85% was obtained when the model reaction was performed in nearly 10 mmol scale, providing promising application in preparative synthesis (Fig. 6a). Then, an intermolecular competition experiment was carried out to explore the selectivity of this oxidative α sp^3^ C–H arylation of alcohols with heteroarenes (see Supplementary Methods). It is significant that the single selectivity and good yield for the oxidative α sp^3^ C–H arylation of alcohols in the presence of ether sp^3^ C–H were observed (Fig. 6b).

## Discussion

**Fig. 7:**
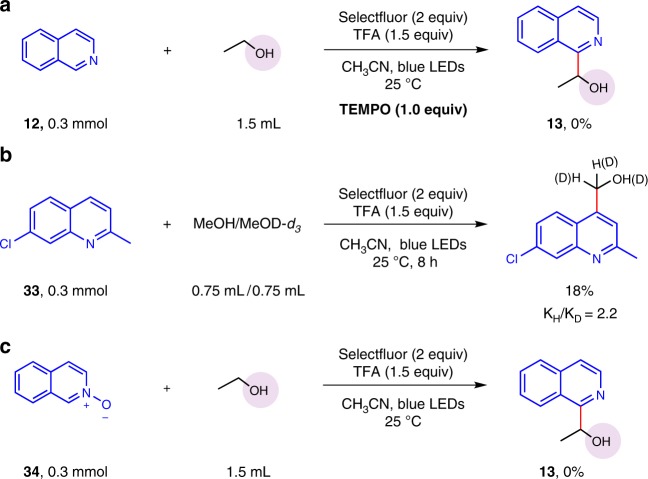
Mechanistic studies. **a** Radical inhibition experiment. **b** Intermolecular kinetic isotope effect experiment. **c** The intermediates experiment

To further understand this visible light-induced protocol, we conducted several mechanistic experiments (Fig. 7). While 2,2,6,6-tetramethylpiperidinooxy (TEMPO) as radical-trapping reagent was subjected to the standard reaction condition (see Supplementary Methods), the oxidative α sp^3^ C–H arylation of alcohols was totally suppressed, thus revealing a radical pathway might be involved (Fig. 7a). Whereafter, the intermolecular kinetic isotope effect (KIE) experiment was undertaken (see Supplementary Methods and Supplementary Fig. 41 for details). A KIE value of 2.2 indicated the cleavage of α sp^3^ C–H is the rate-determining step for this protocol (Fig. 7b). Importantly, the highly reactive *N*-oxide is not the reaction intermediate^59^, because no product was afforded while isoquinoline *N*-oxide **34** was employed (Fig. 7c).

In summary, we have developed a visible light-induced oxidative α sp^3^ C–H arylation of alcohols with heteroarenes, which is promoted by selectfluor under the blue LEDs irradiation. What is essential for this protocol is the N–F activation of selectfluor achieved by blue light irradiation. The EPR study provided important evidence for the visible light-induced N–F activation of selectfluor. The selective oxidative α sp^3^ C–H arylation of alcohols with heteroarenes in the presence of ethers is demonstrated. The development of related oxidative C(sp^3^)–H functionalization is underway in our laboratory.

## Methods

### General procedure (**13**)

A solution of isoquinoline **12** (0.3 mmol, 1.0 equiv, 38.7 mg), 1.5 mL ethanol, selectfluor (0.6 mmol, 2.0 equiv, 212.5 mg) and TFA (0.45 mmol, 1.5 equiv, 51.3 mg) in degassed dry CH_3_CN (2.0 mL) were stirred under nitrogen atmosphere and irradiated by 3 W blue LEDs at 25 °C for 24 h. Afterwards, the reaction system was quenched by saturated NaHCO_3_ aqueous solution. The aqueous solution was extracted with ethyl acetate (3 × 10 mL) and the combined extracts were dried with anhydrous Na_2_SO_4_. The solvents were removed under reduced pressure by rotary evaporation. Then, the pure product was obtained by flash column chromatography on silica gel (eluent: petroleum ether/ethyl acetate = 5:1), directly giving the desired product **13** in 87% yield as a pale yellow liquid. For ^1^H NMR and ^13^C NMR spectra of compounds **13**–**32** see Supplementary Figs. 1–40. Full experimental details can be found in the Supplementary Methods.

## Supplementary information

Supplementary Information

## Data Availability

The authors declare that the data supporting the findings of this study are available within the article and its Supplementary information files.
