# Supplementary material for: Visible light-induced direct α C–H functionalization of alcohols
Source: Nat Commun. 2019 Jan 28;10:467. doi: 10.1038/s41467-019-08413-9 (PMC6349847; doi:10.1038/s41467-019-08413-9)
Supplement: Supplementary file 1 — Supplementary Information [file 41467_2019_8413_MOESM1_ESM.pdf]

Supplementary Information

Visible Light-Induced Direct α C–H Functionalization of Alcohols

Niu et al.

Supplementary Figures

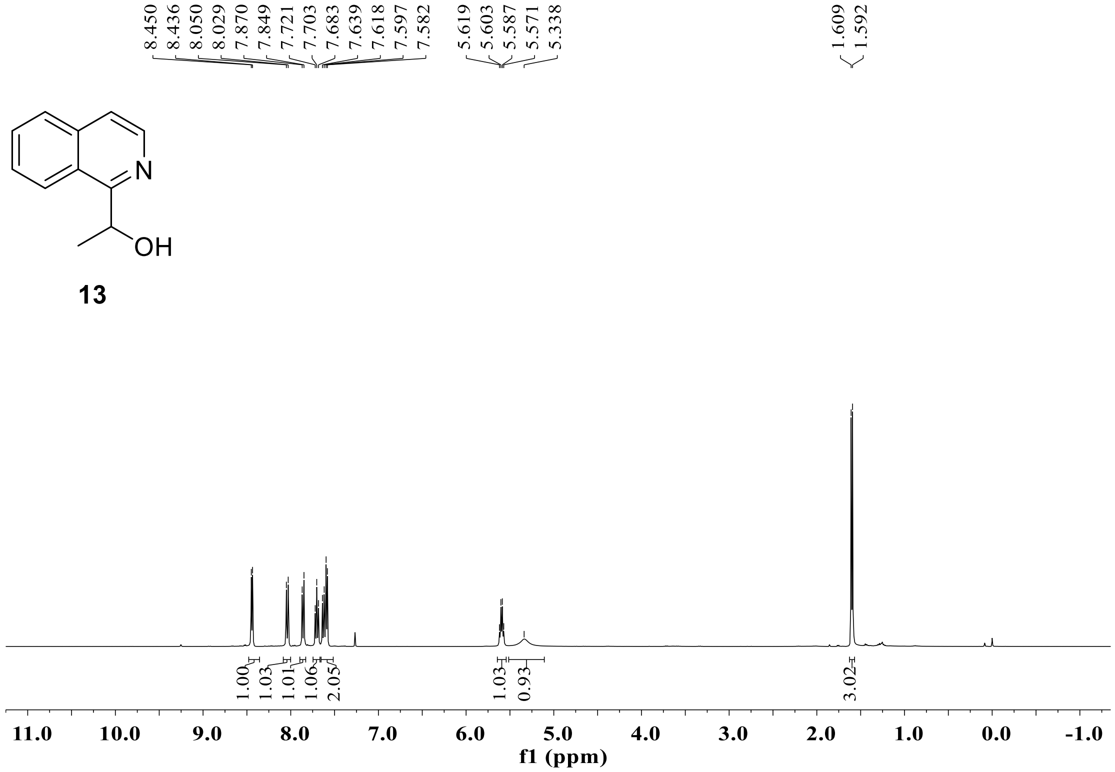

Supplementary Figure 1. ^{1}H **NMR (400 MHz, $CDCl_3$) spectrum of 13**

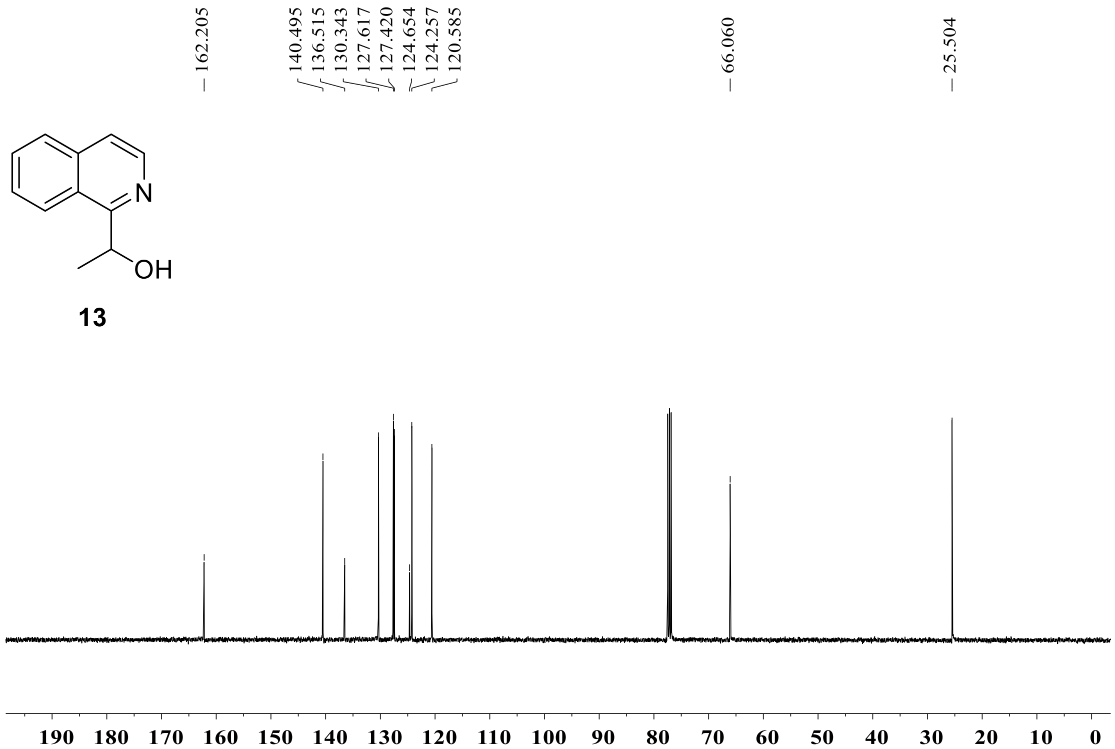

Supplementary Figure 2. ^{13}C **NMR (101MHz, $CDCl_3$) spectrum of 13**

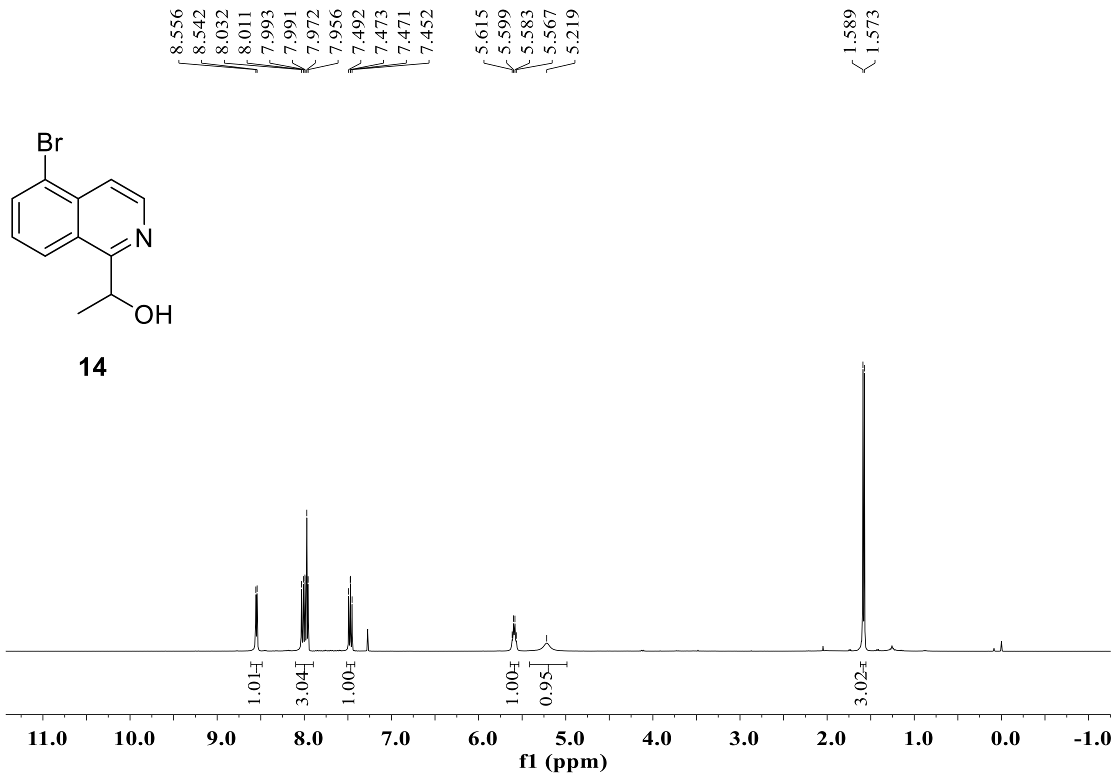

Supplementary Figure 3. ^{1}**H NMR (400 MHz, CDCl$_3$) spectrum of 14**

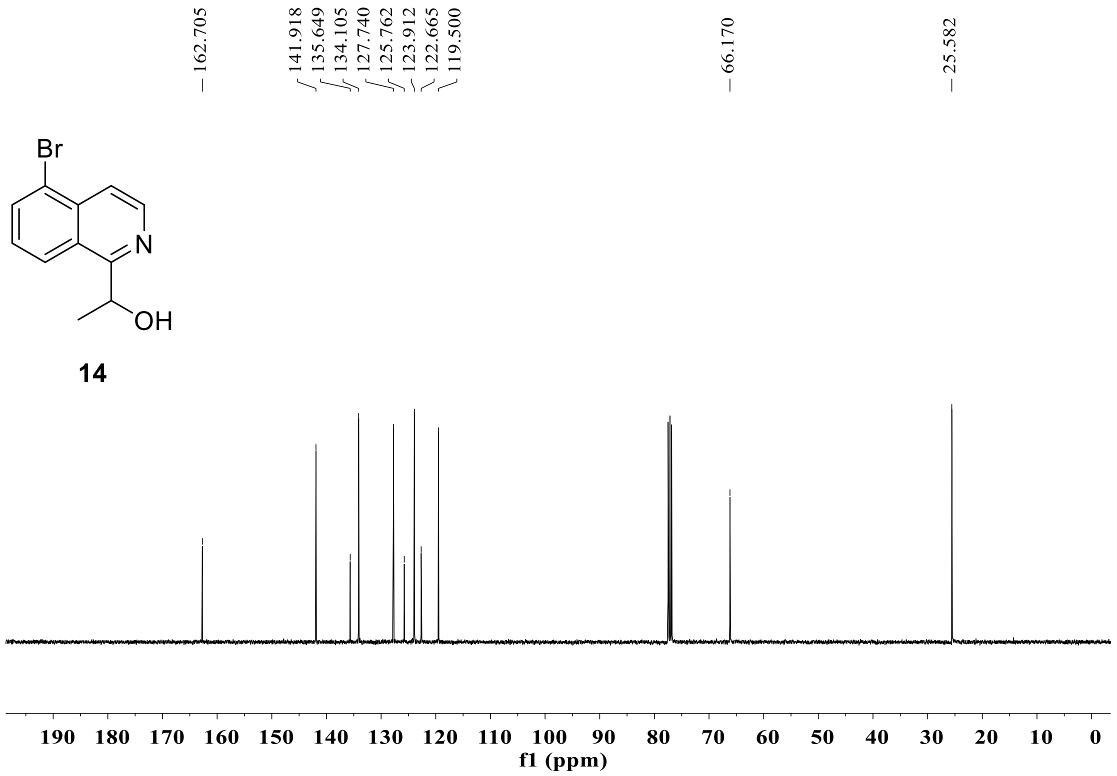

Supplementary Figure 4. ^{13}**C NMR (101MHz, CDCl$_3$) spectrum of 14**

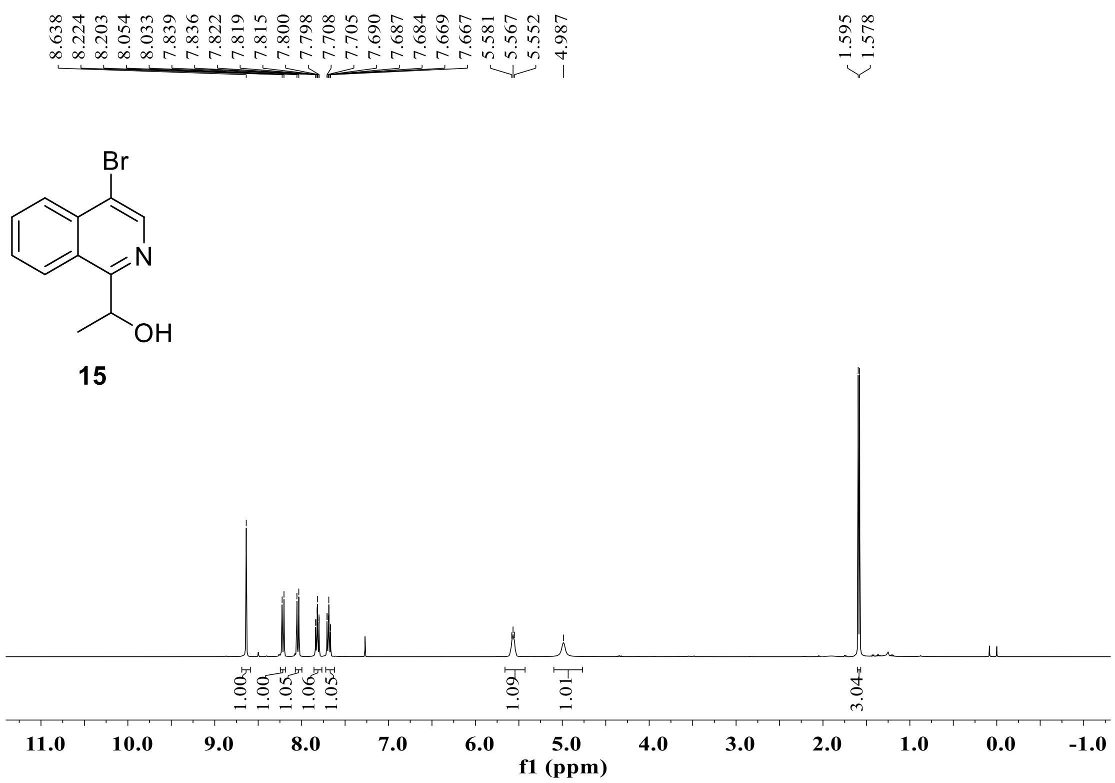

Supplementary Figure 5. ^{1}**H NMR (400 MHz,** $CDCl_3$**) spectrum of 15**

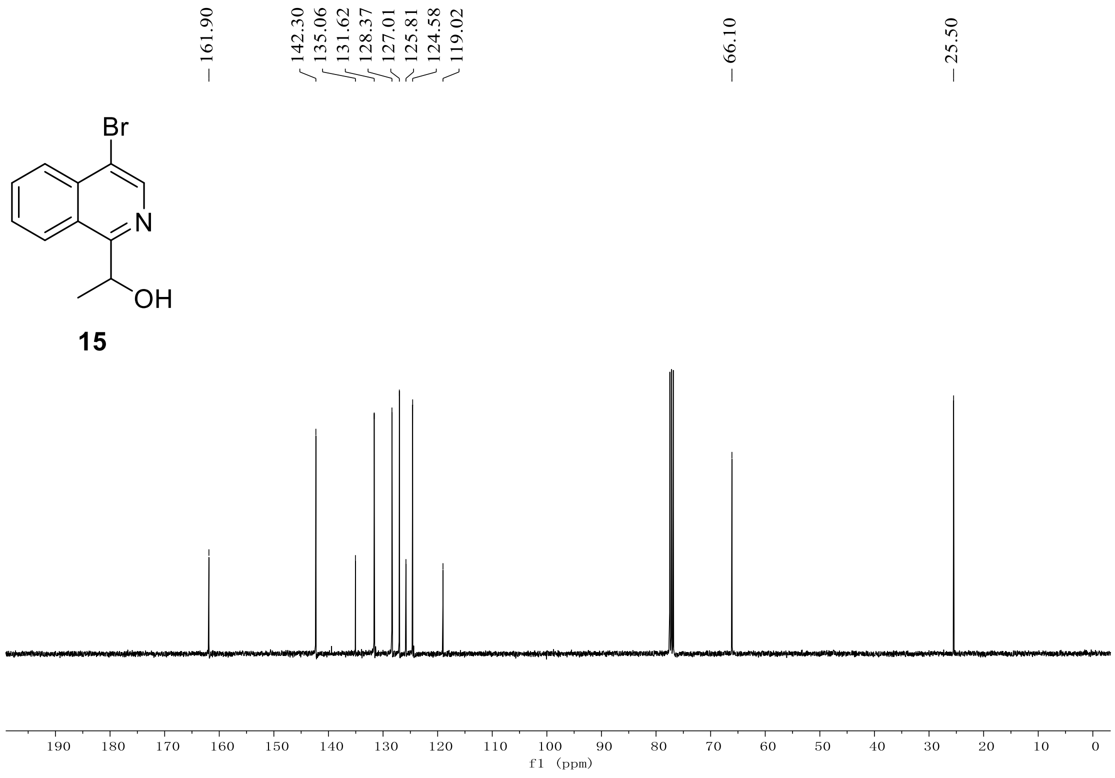

Supplementary Figure 6. ^{13}**C NMR (101MHz,** $CDCl_3$**) spectrum of 15**

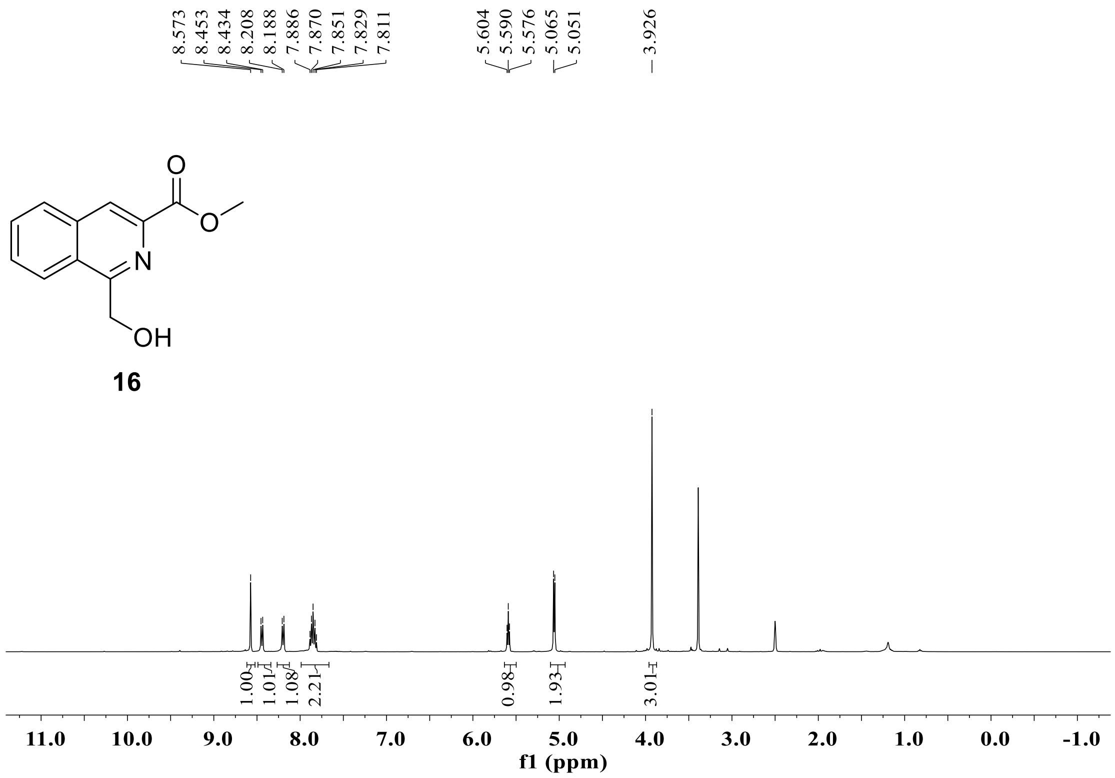

Supplementary Figure 7. ^{1}**H NMR (400 MHz, DMSO-d_6) spectrum of 16**

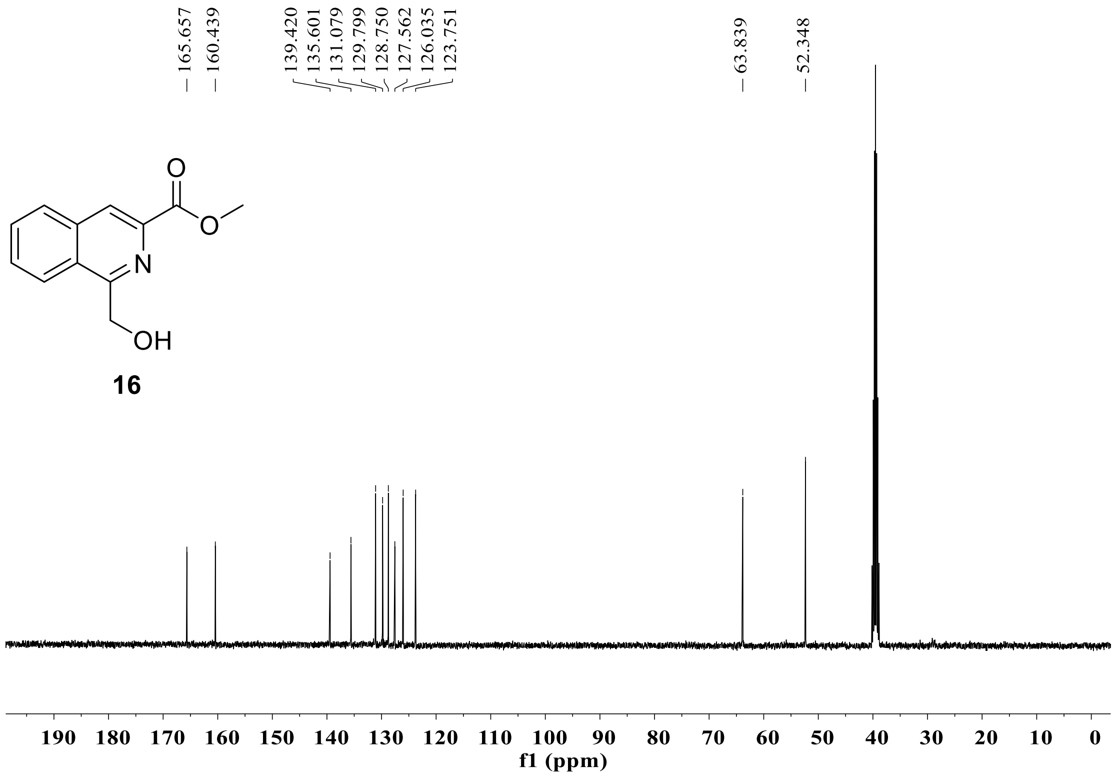

Supplementary Figure 8. ^{13}**C NMR (101MHz, DMSO-d_6) spectrum of 16**

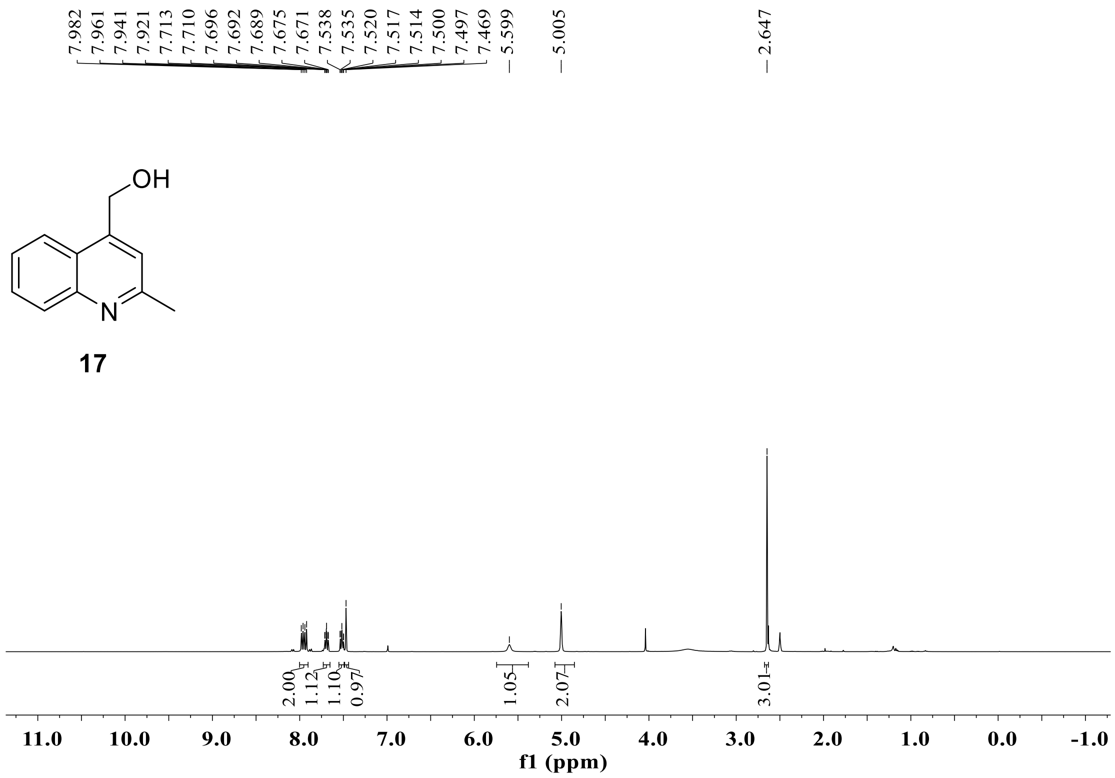

Supplementary Figure 9. ^{1}H NMR (400 MHz, DMSO-d_6) spectrum of 17

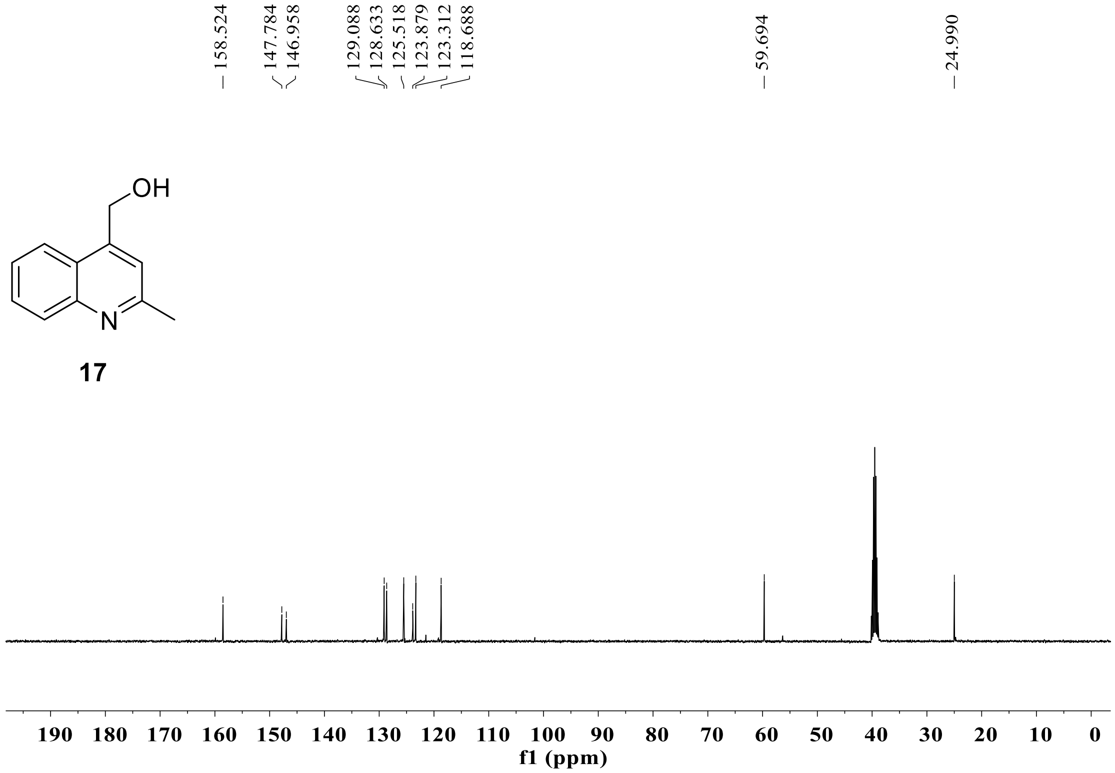

Supplementary Figure 10. ^{13}C NMR (101MHz, DMSO-d_6) spectrum of 17

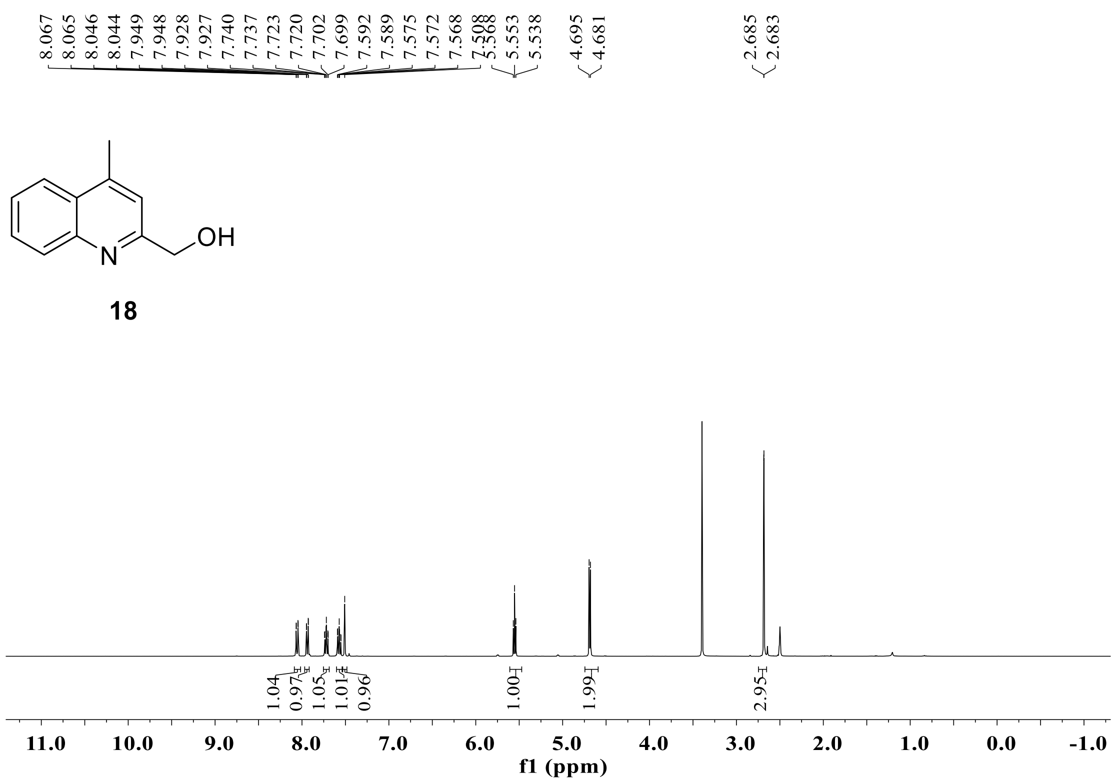

Supplementary Figure 11. ^{1}H NMR (400 MHz, DMSO-d_6) spectrum of 18

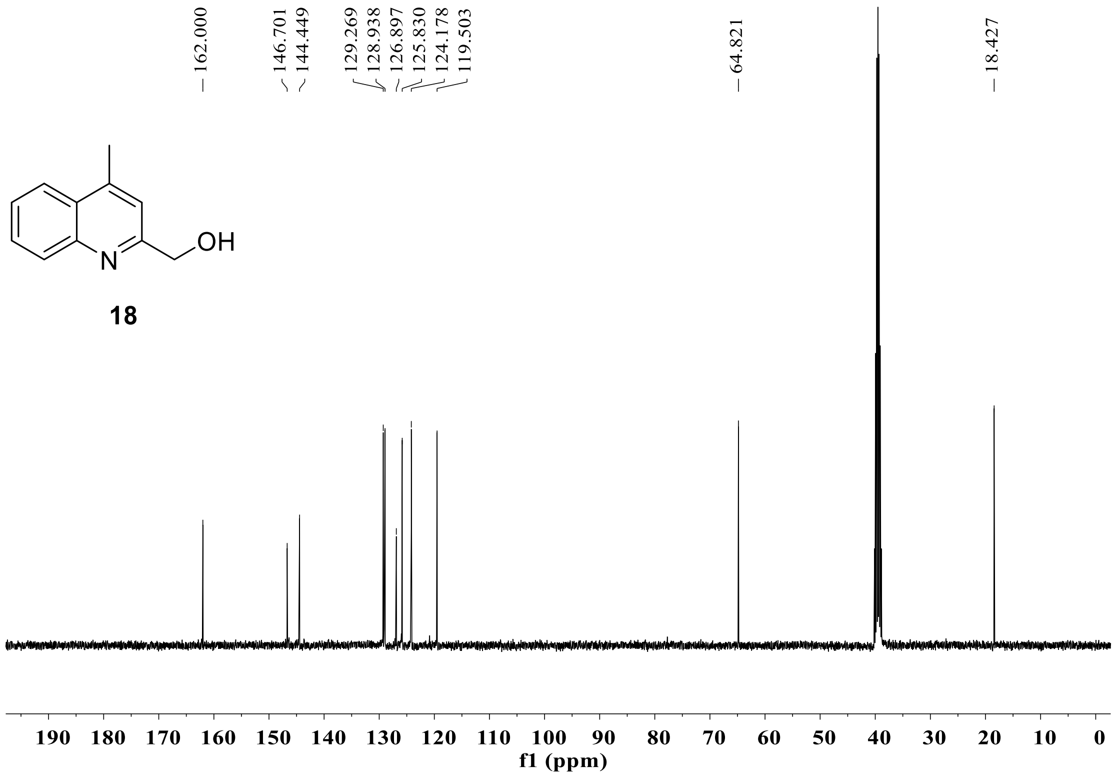

Supplementary Figure 12. ^{13}C NMR (101MHz, DMSO-d_6) spectrum of 18

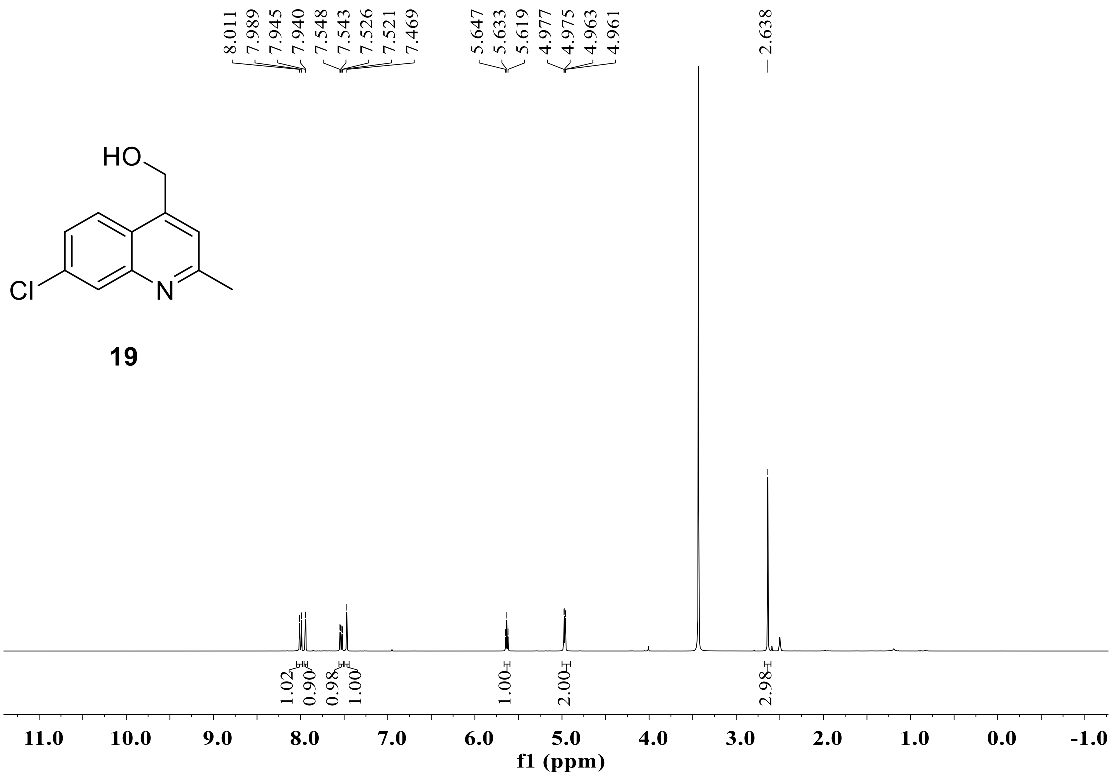

Supplementary Figure 13. ^{1}H NMR (400 MHz, DMSO-d_6) spectrum of 19

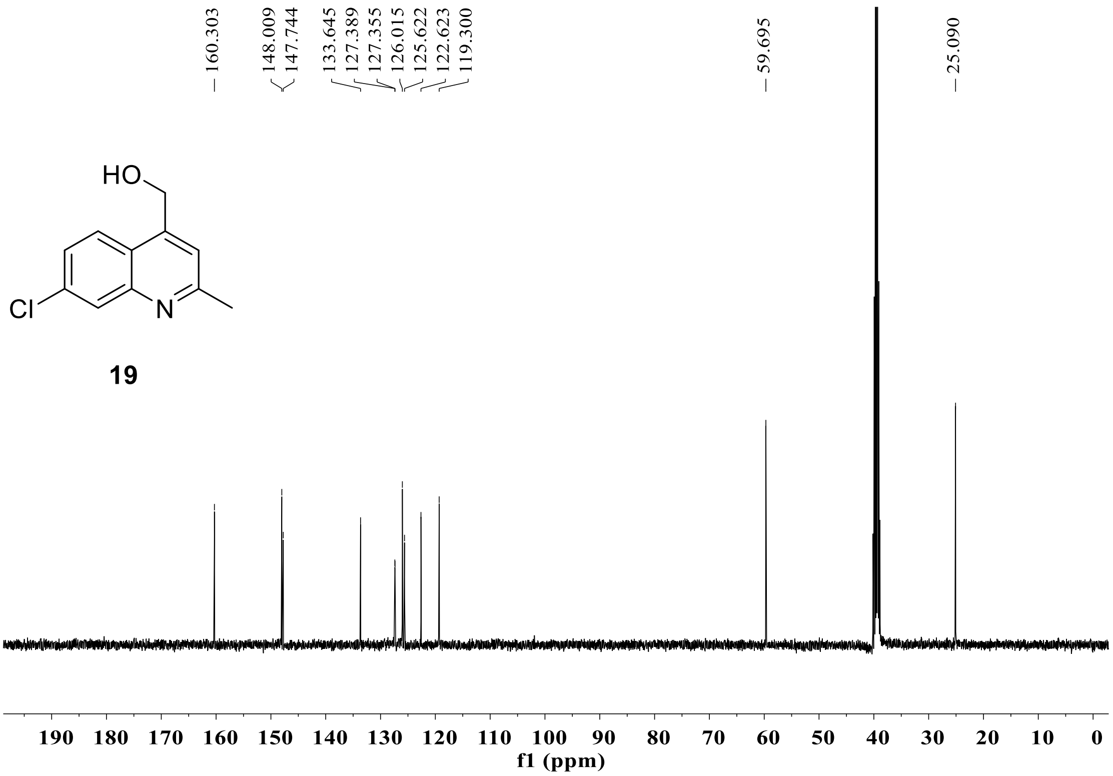

Supplementary Figure 14. ^{13}C NMR (101MHz, DMSO-d_6) spectrum of 19

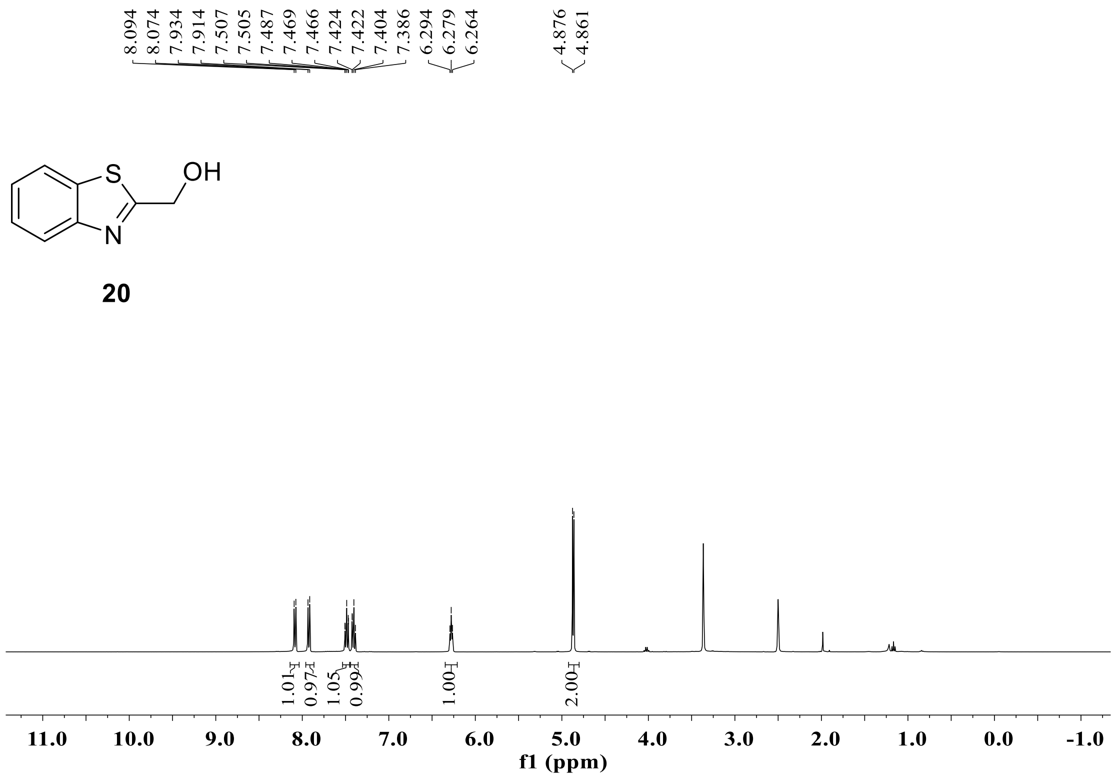

Supplementary Figure 15. ^{1}**H NMR (400 MHz, DMSO-*d*$_6$) spectrum of 20**

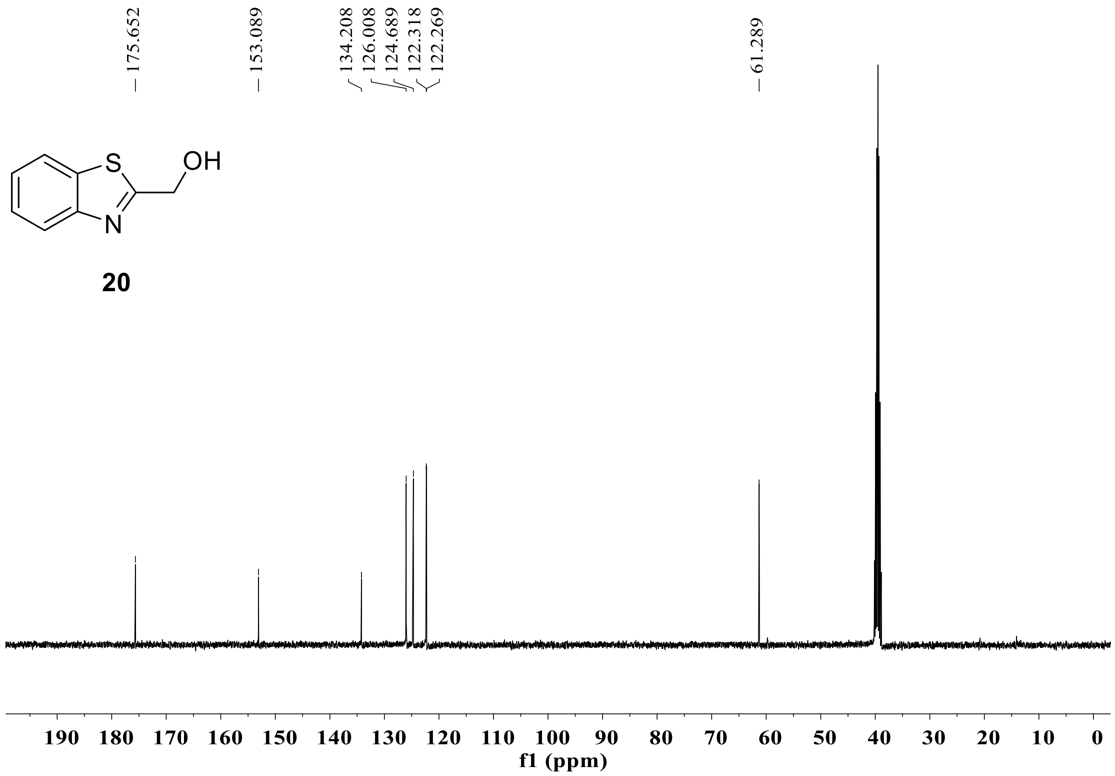

Supplementary Figure 16. ^{13}**C NMR (101MHz, DMSO-*d*$_6$) spectrum of 20**

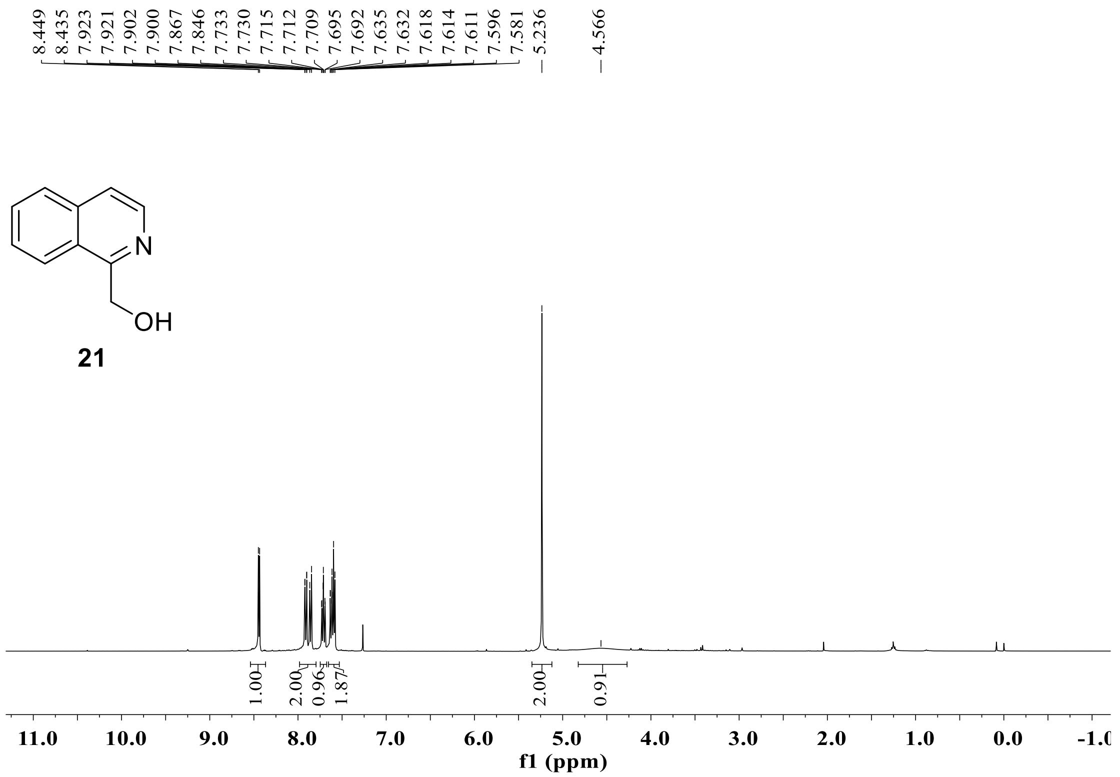

Supplementary Figure 17. ^{1}H **NMR (400 MHz,** $CDCl_3$**) spectrum of 21**

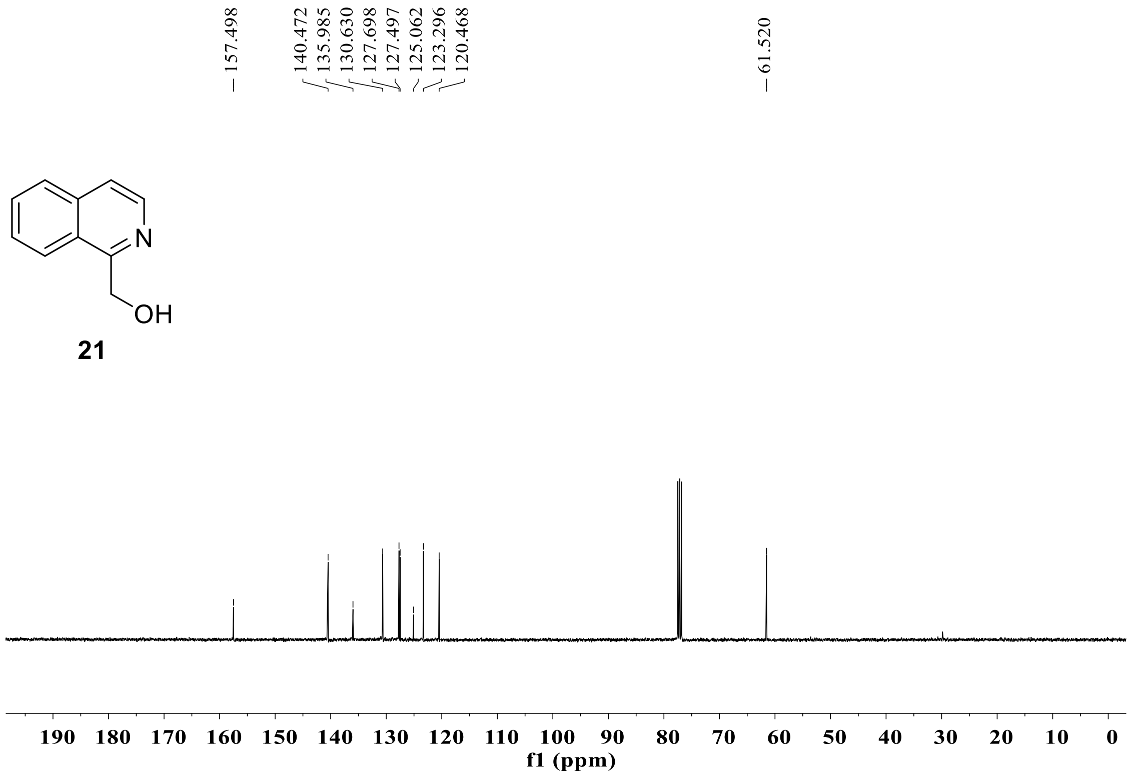

Supplementary Figure 18. ^{13}C **NMR (101MHz,** $CDCl_3$**) spectrum of 21**

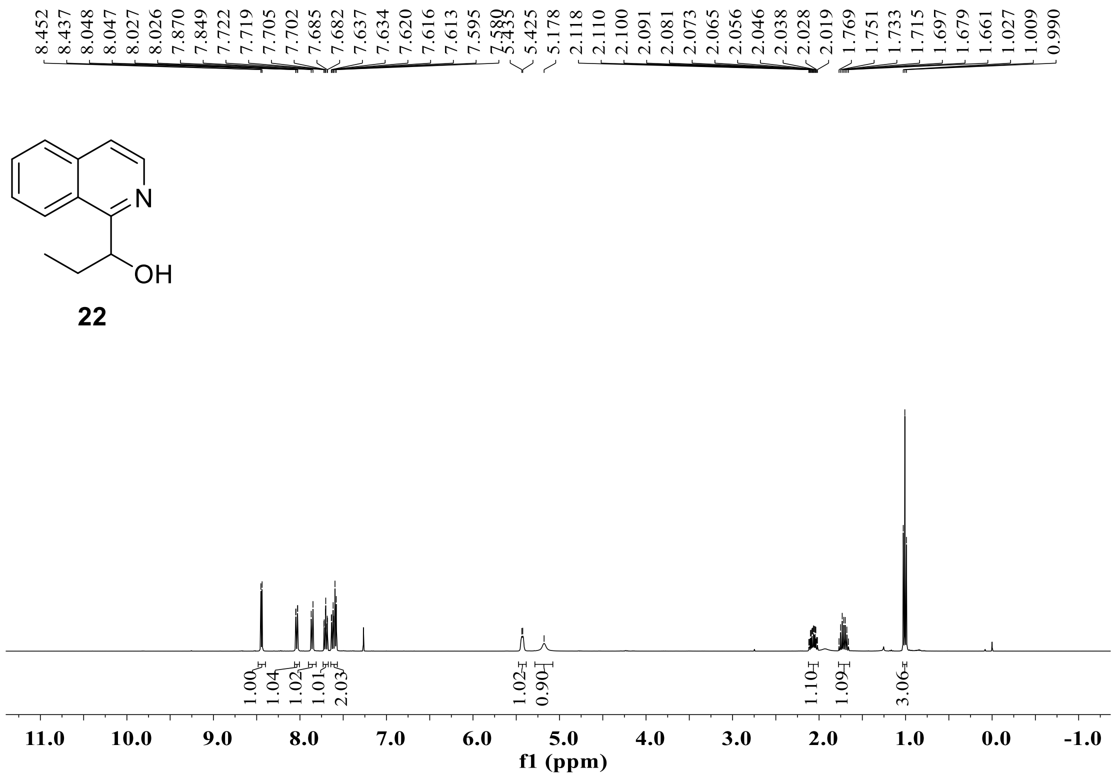

Supplementary Figure 19. ^{1}H NMR (400 MHz, $CDCl_3$) spectrum of 22

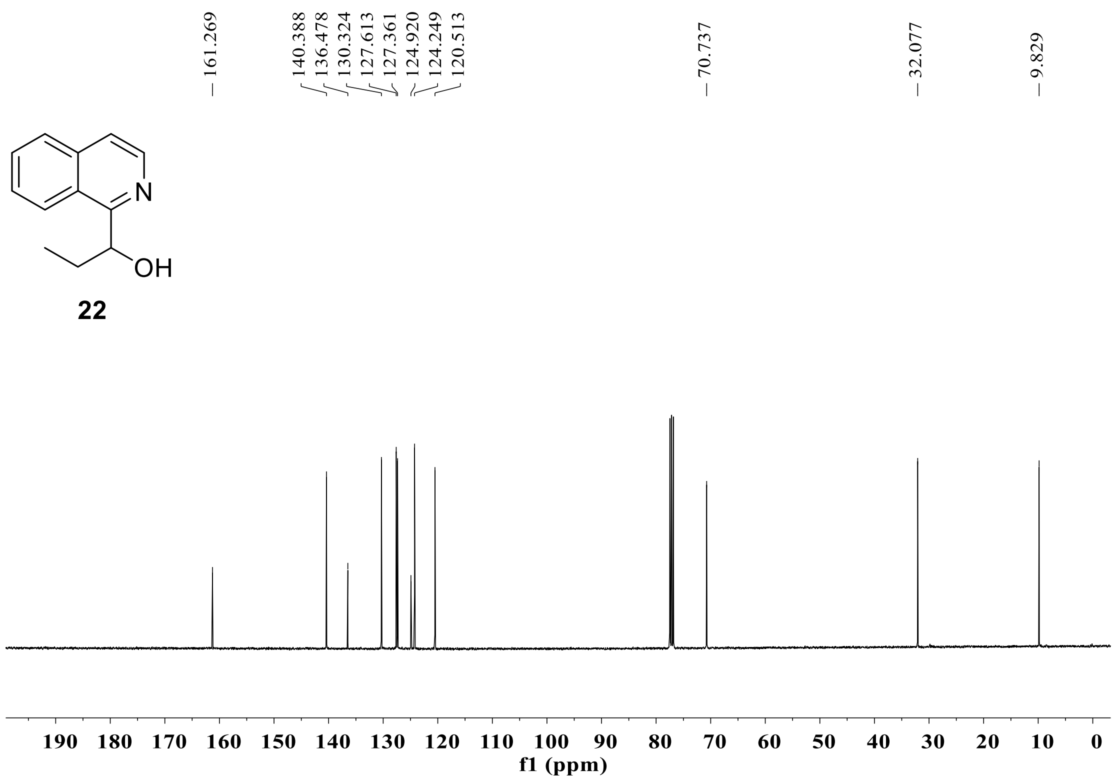

Supplementary Figure 20. ^{13}C NMR (101MHz, $CDCl_3$) spectrum of 22

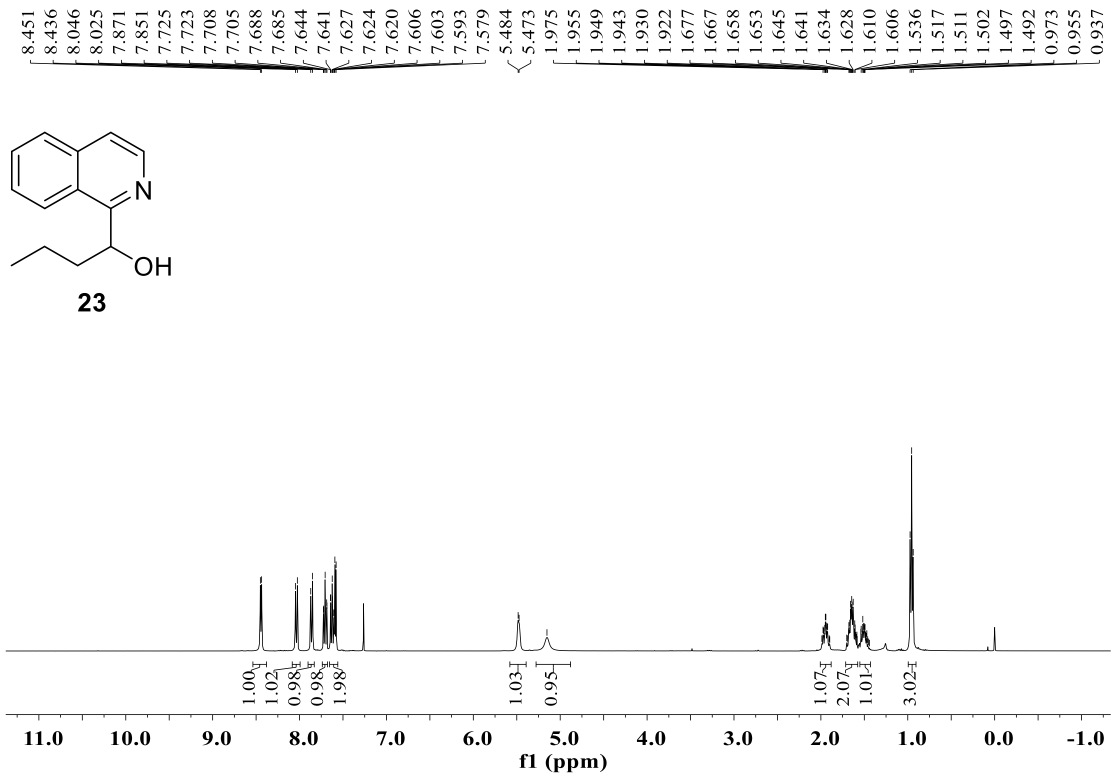

Supplementary Figure 21. ^{1}H NMR (400 MHz, $CDCl_3$) spectrum of 23

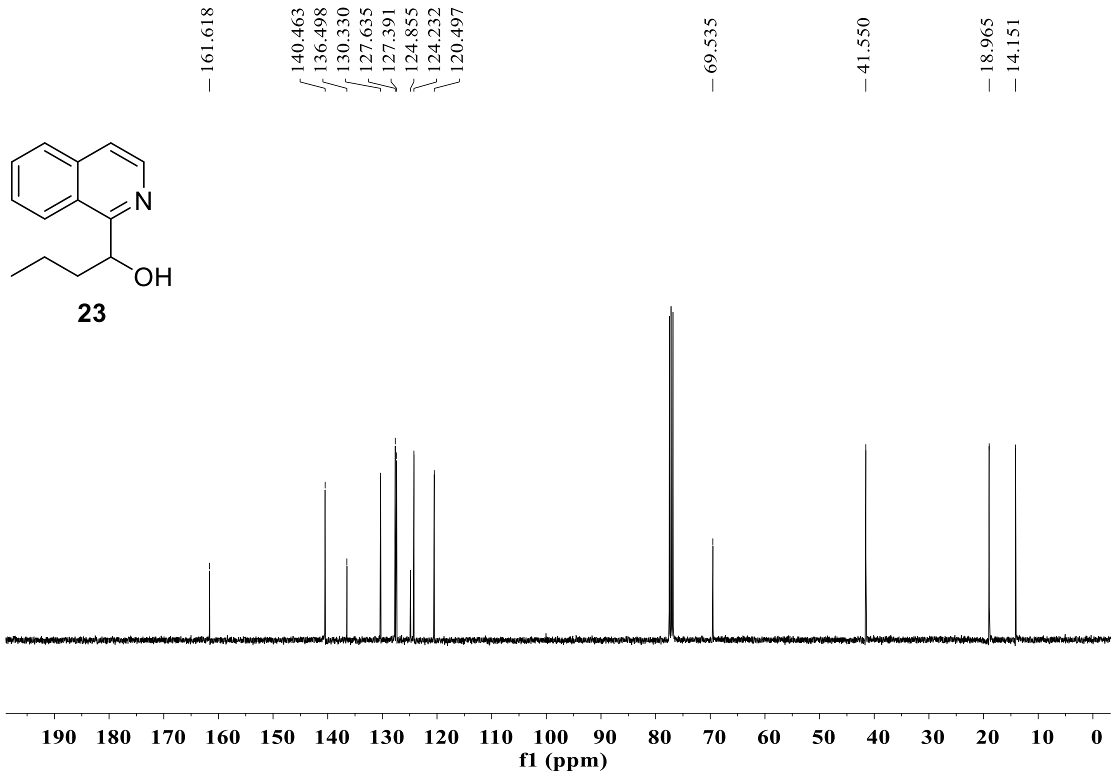

Supplementary Figure 22. ^{13}C NMR (101MHz, $CDCl_3$) spectrum of 23

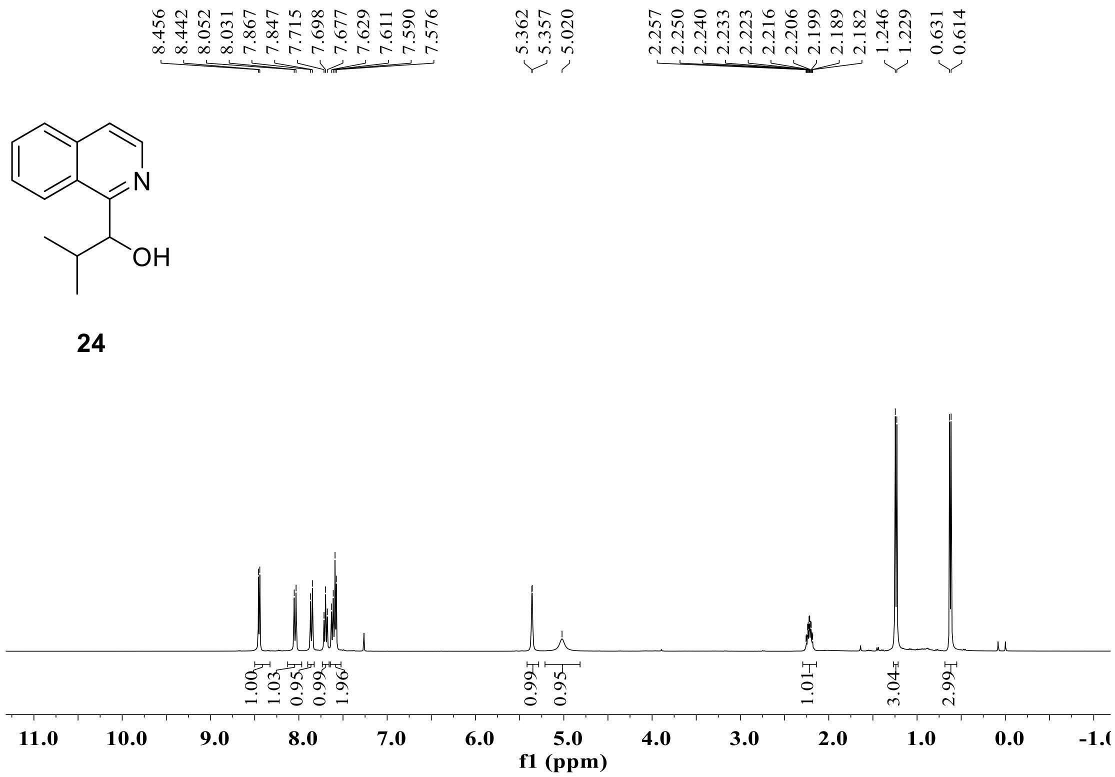

Supplementary Figure 23. ^{1}H NMR (400 MHz, $CDCl_3$) spectrum of 24

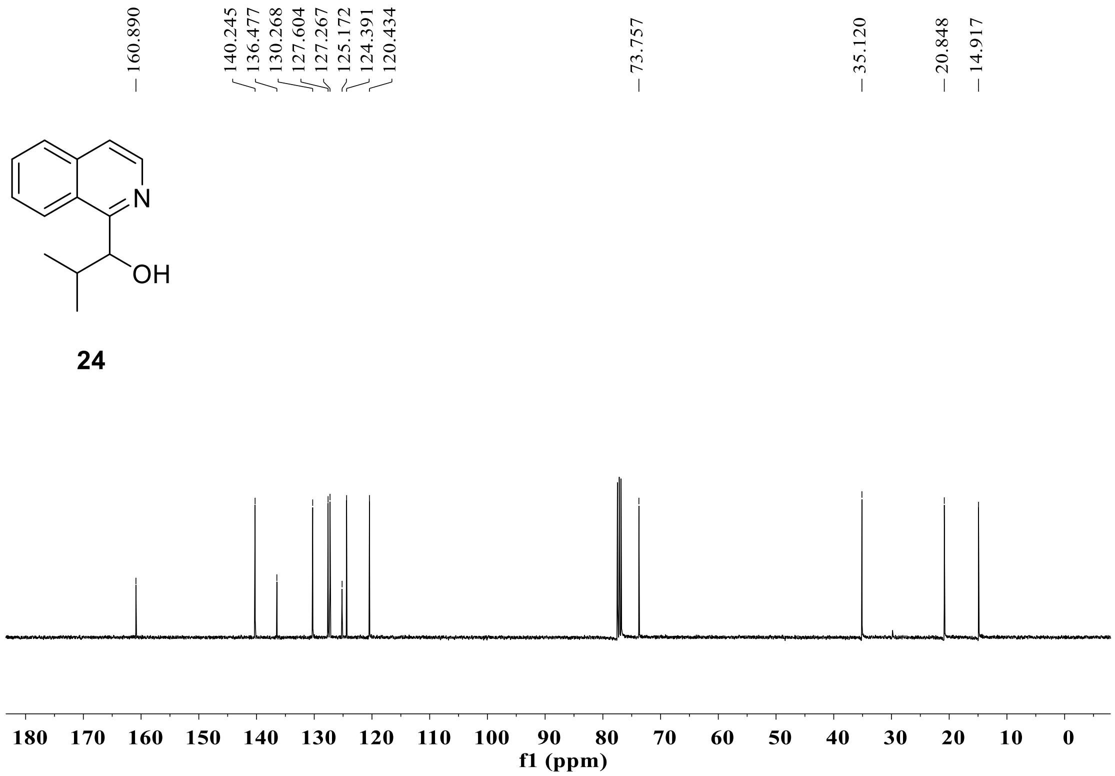

Supplementary Figure 24. ^{13}C NMR (101MHz, $CDCl_3$) spectrum of 24

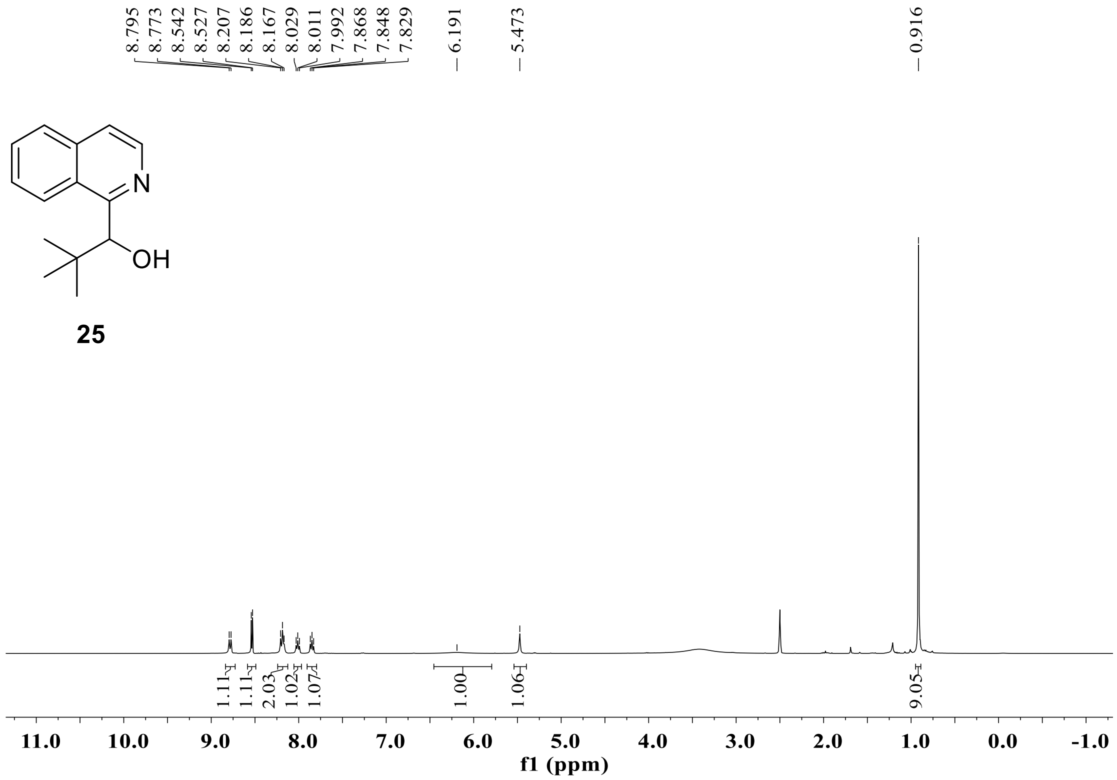

Supplementary Figure 25. ^{1}**H NMR (400 MHz, DMSO-*d*$_6$) spectrum of 25**

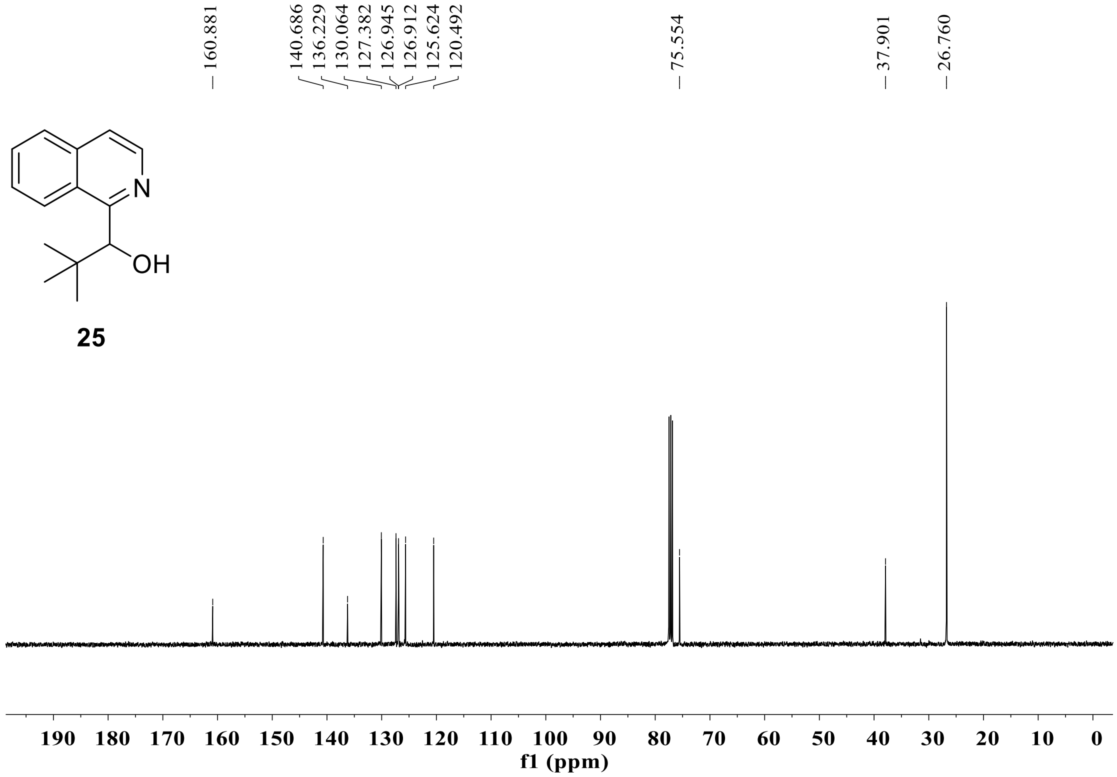

Supplementary Figure 26. ^{13}**C NMR (101MHz, $CDCl_3$) spectrum of 25**

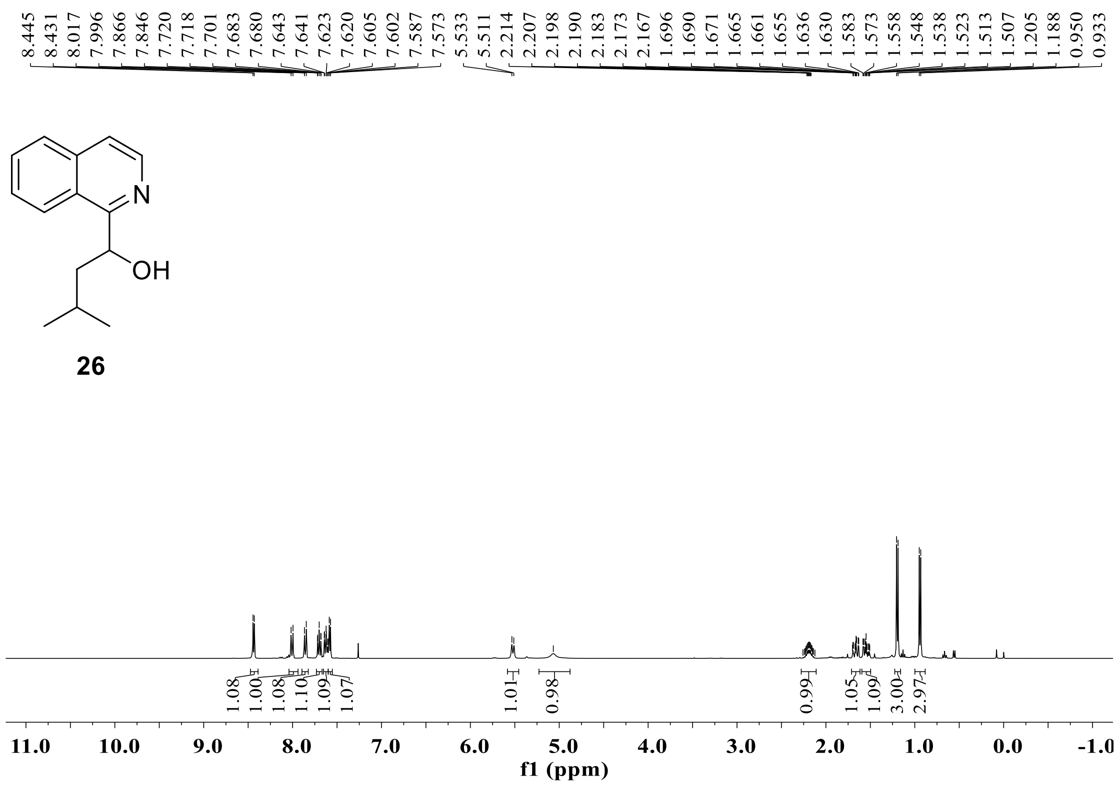

Supplementary Figure 27. ^{1}H NMR (400 MHz, $CDCl_3$) spectrum of 26

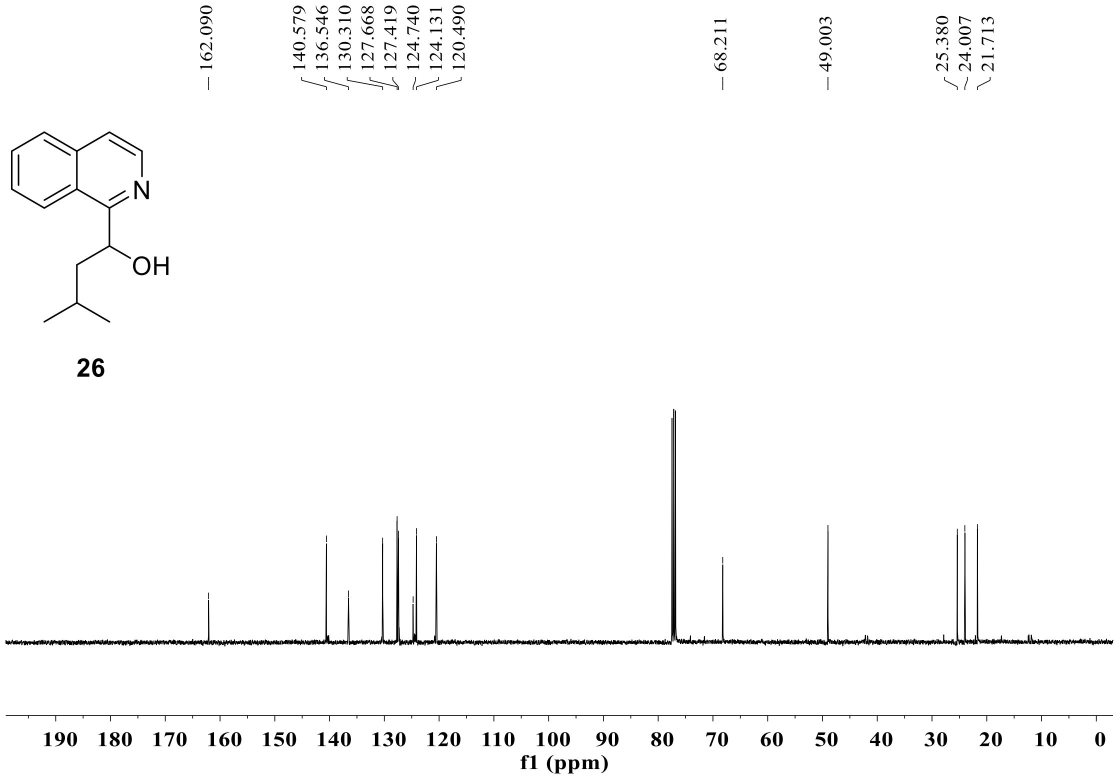

Supplementary Figure 28. ^{13}C NMR (101MHz, $CDCl_3$) spectrum of 26

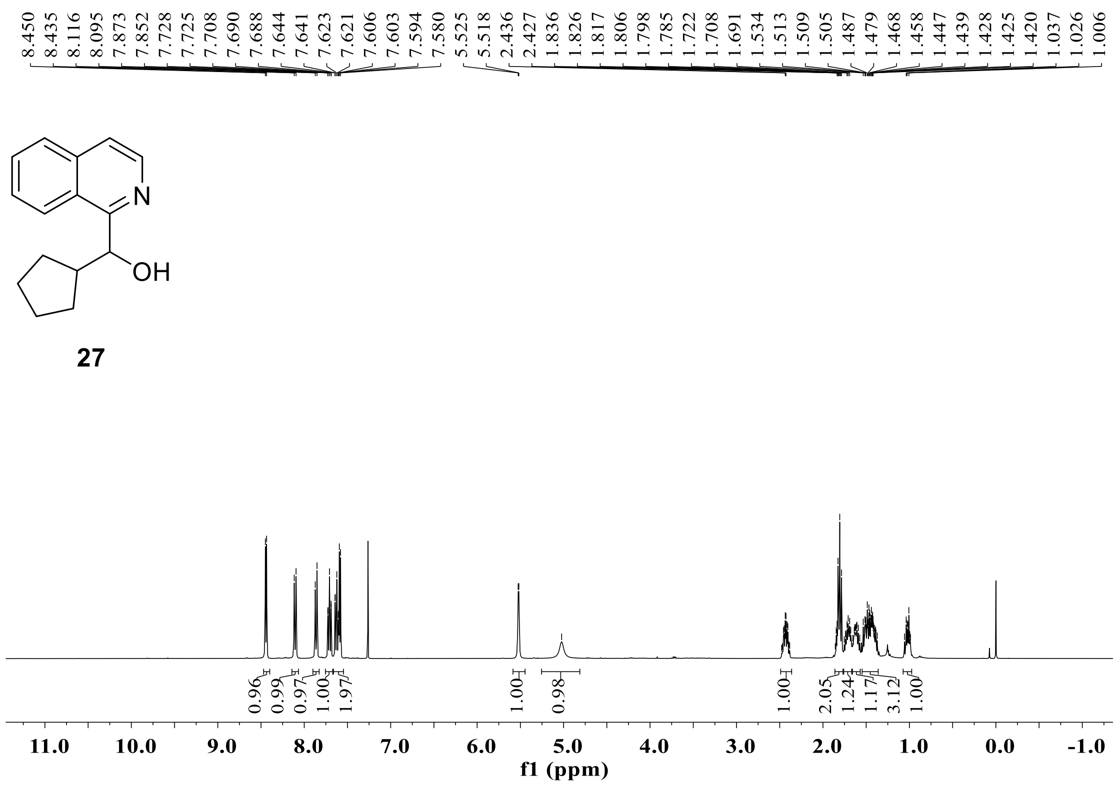

Supplementary Figure 29. ^{1}H NMR (400 MHz, $CDCl_3$) spectrum of 27

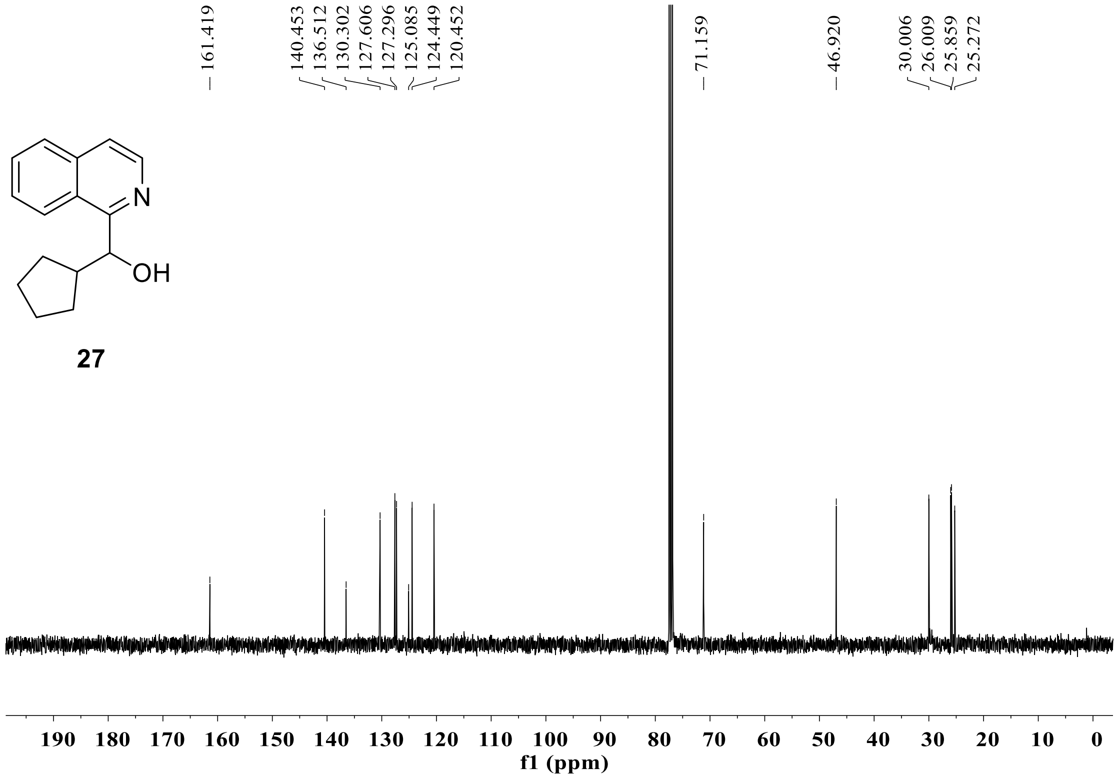

Supplementary Figure 30. ^{13}C NMR (101MHz, $CDCl_3$) spectrum of 27

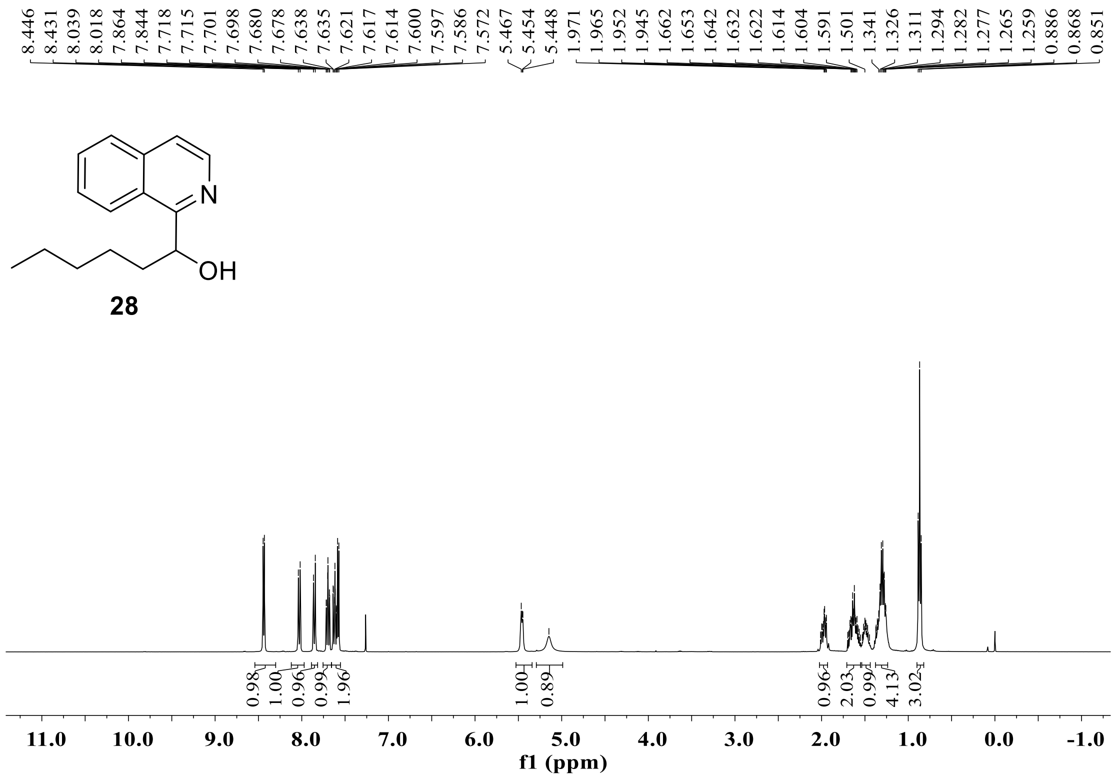

Supplementary Figure 31. ^{1}H NMR (400 MHz, $CDCl_3$) spectrum of 28

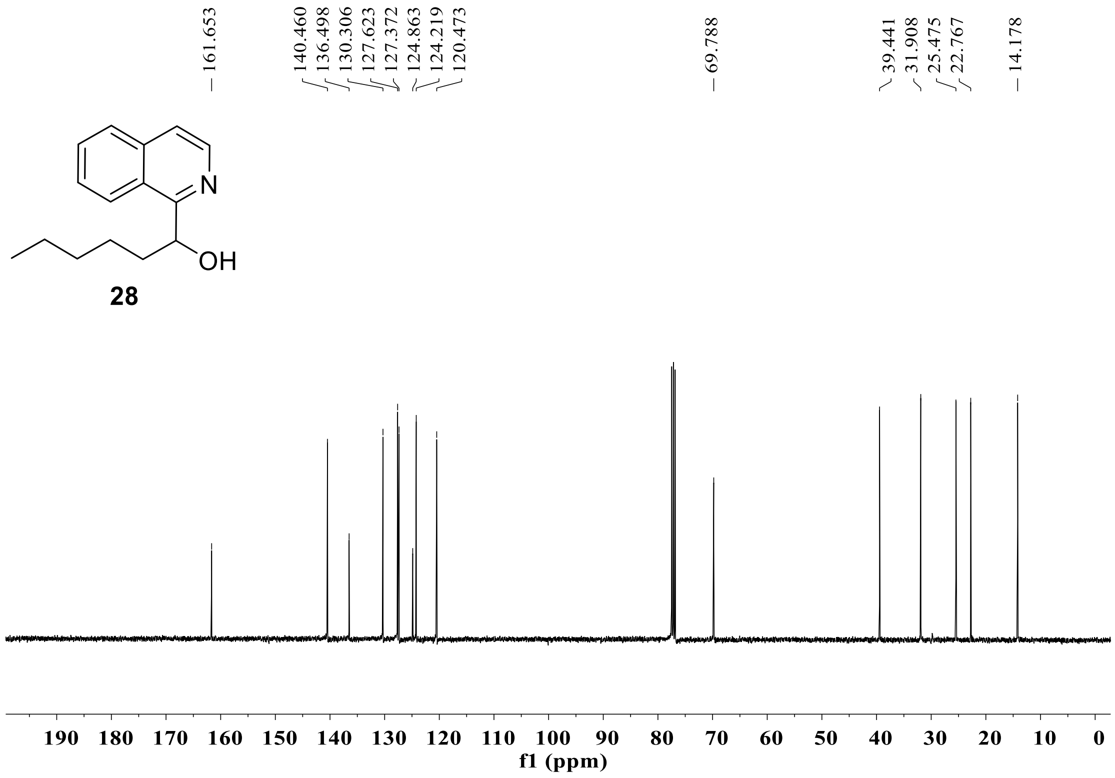

Supplementary Figure 32. ^{13}C NMR (101MHz, $CDCl_3$) spectrum of 28

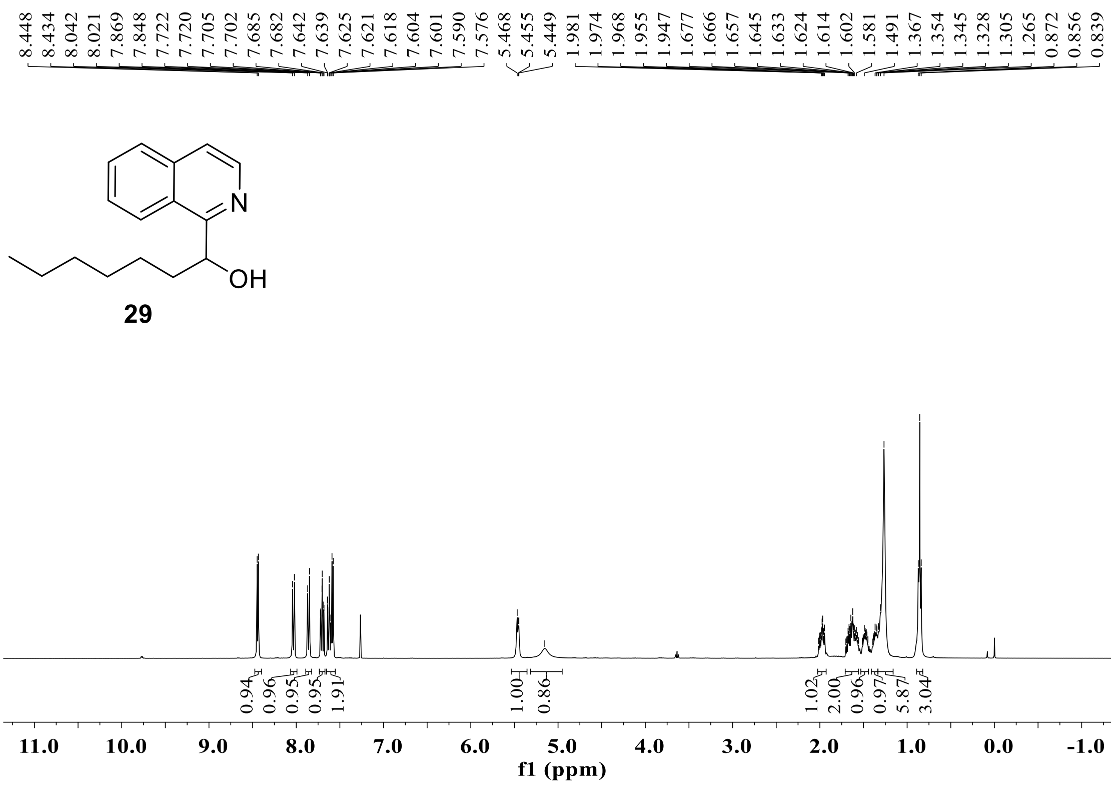

Supplementary Figure 33. ^{1}H **NMR (400 MHz,** $CDCl_3$**) spectrum of 29**

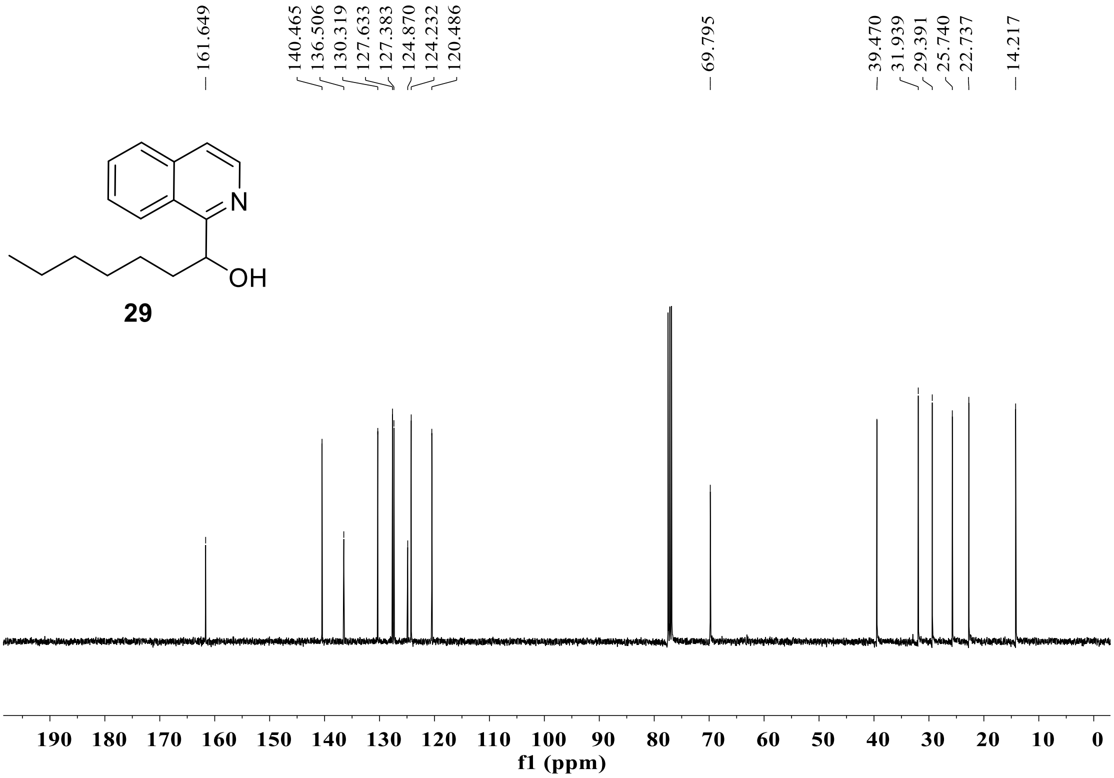

Supplementary Figure 34. ^{13}C **NMR (101MHz,** $CDCl_3$**) spectrum of 29**

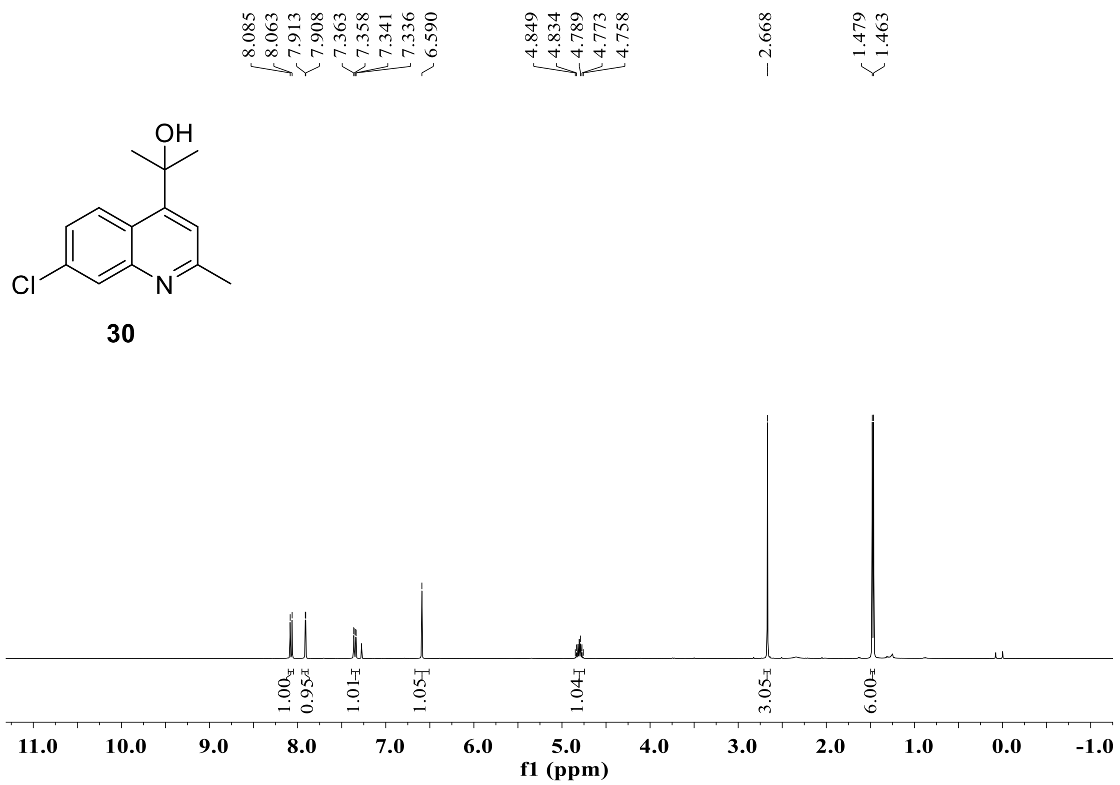

Supplementary Figure 35. **^{1}H NMR (400 MHz, $CDCl_3$) spectrum of 30**

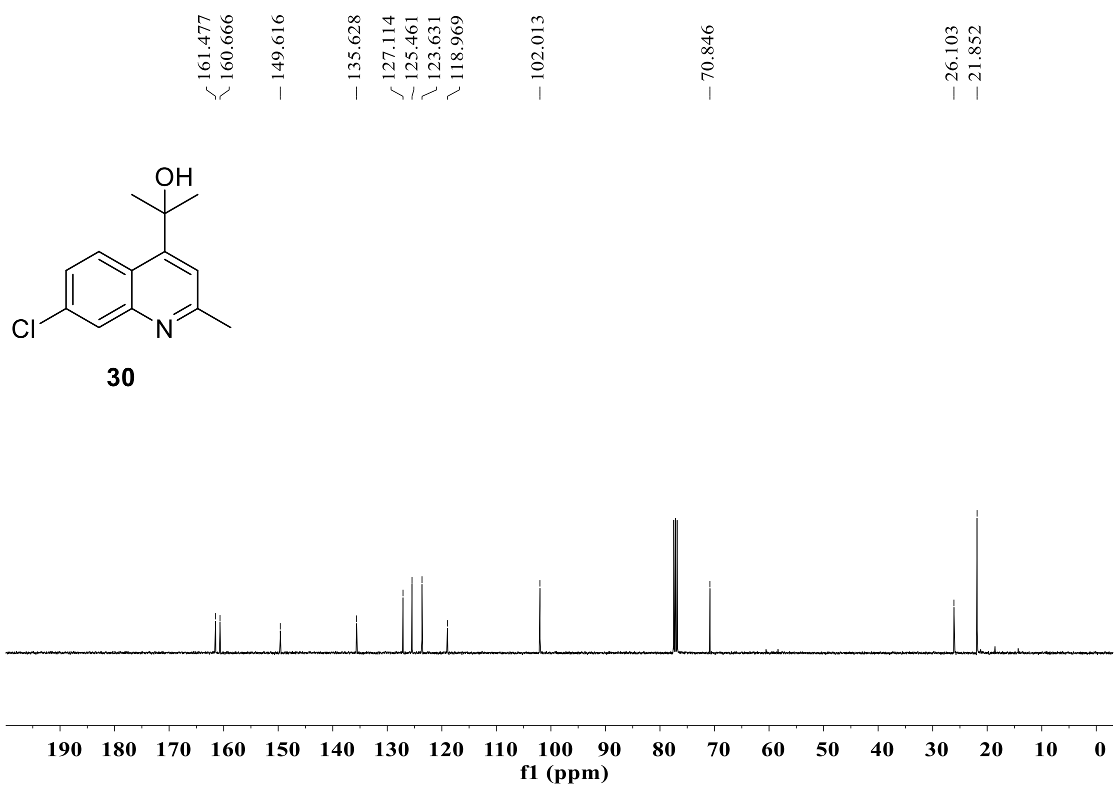

Supplementary Figure 36. **^{13}C NMR (101MHz, $CDCl_3$) spectrum of 30**

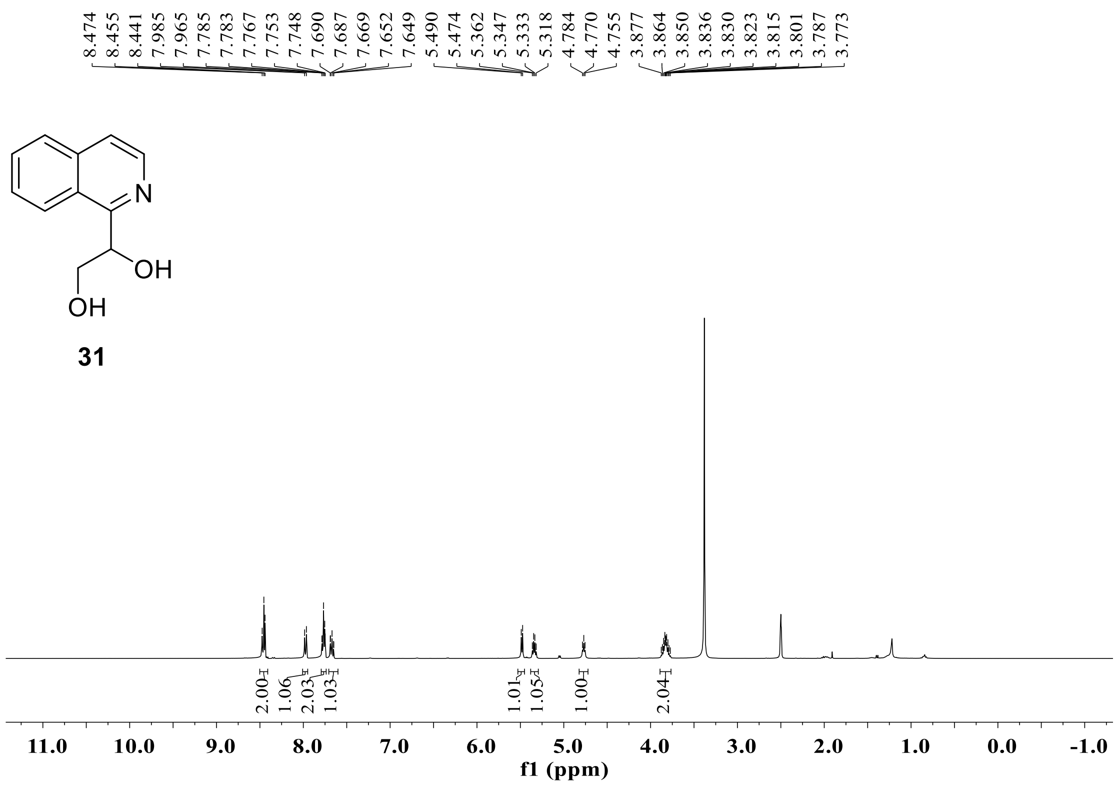

Supplementary Figure 37. ^{1}H **NMR (400 MHz, DMSO-d_6) spectrum of 31**

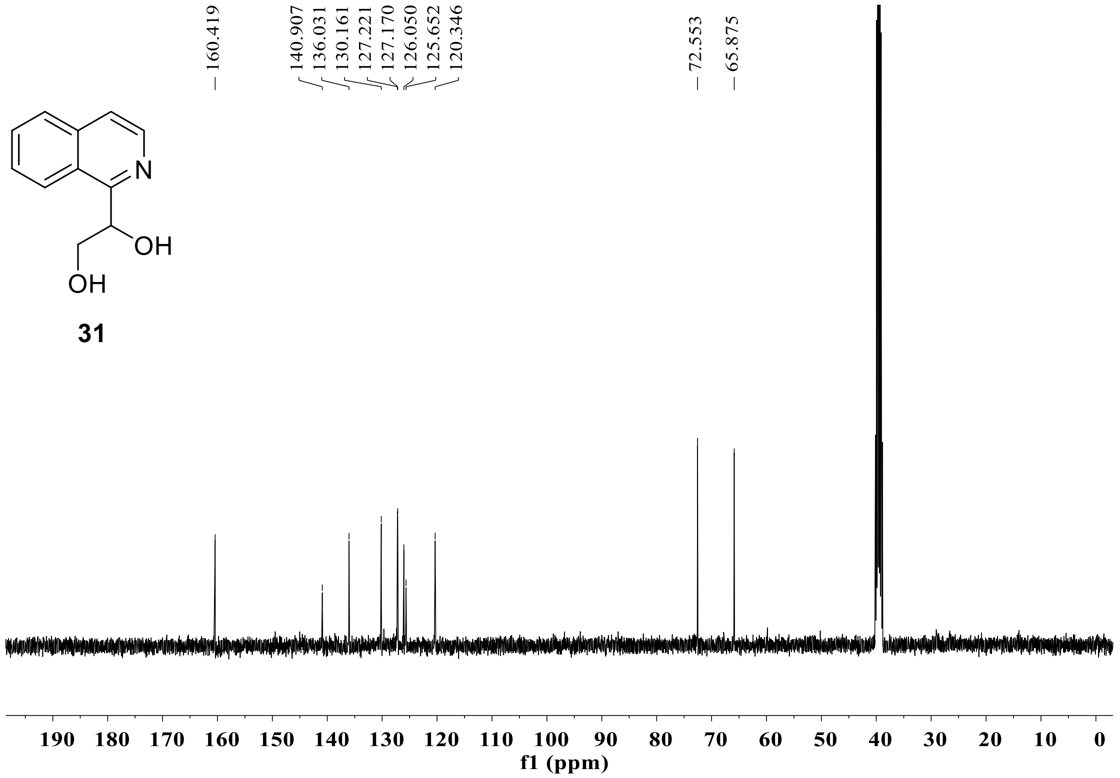

Supplementary Figure 38. ^{13}C **NMR (101MHz, DMSO-d_6) spectrum of 31**

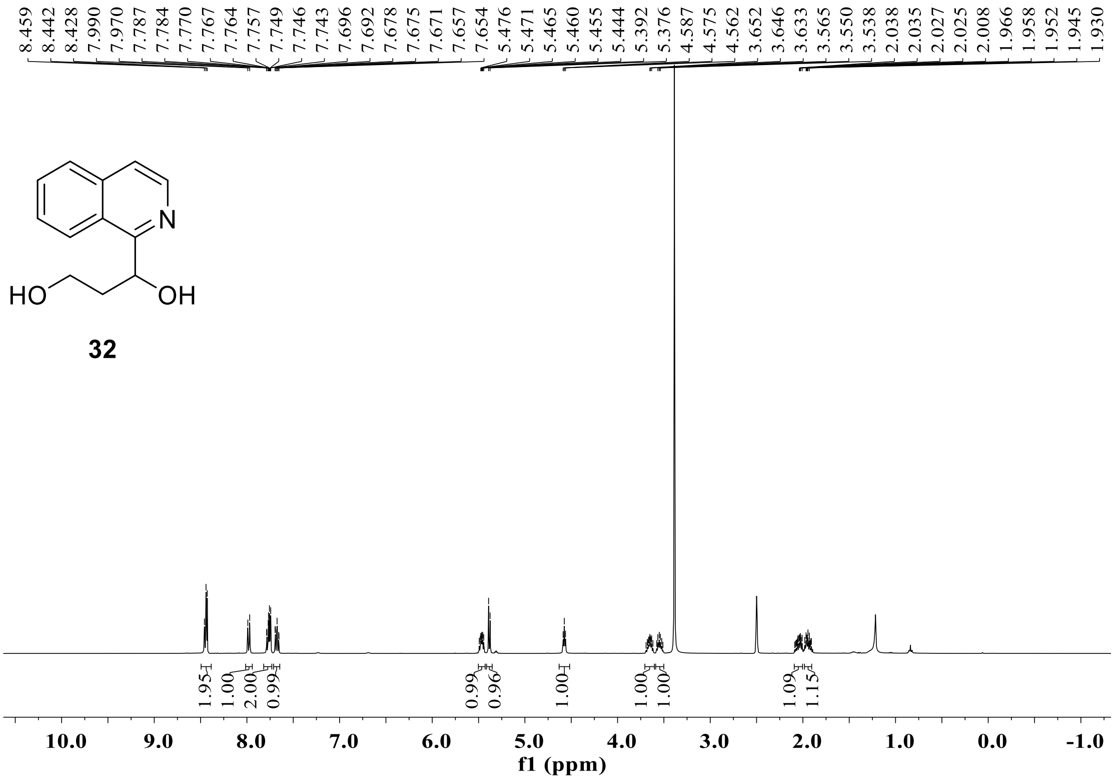

Supplementary Figure 39. ^{1}H **NMR (400 MHz, DMSO-d_6) spectrum of 32**

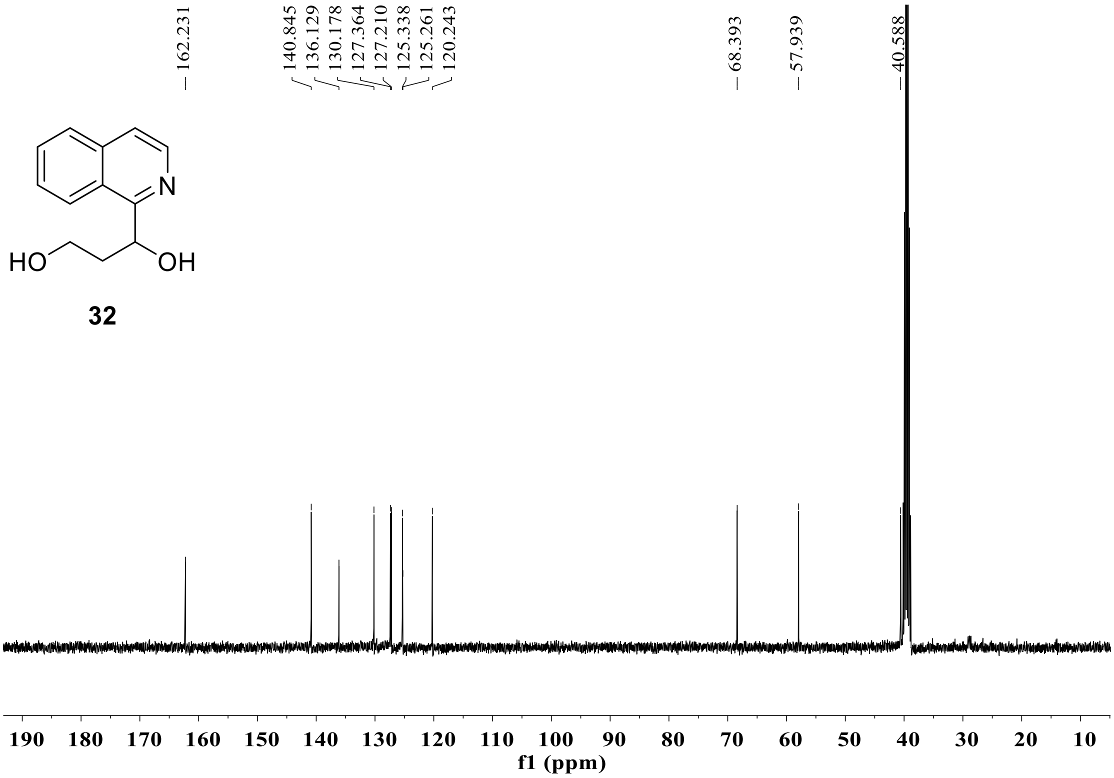

Supplementary Figure 40. ^{13}C **NMR (101MHz, DMSO-d_6) spectrum of 32**

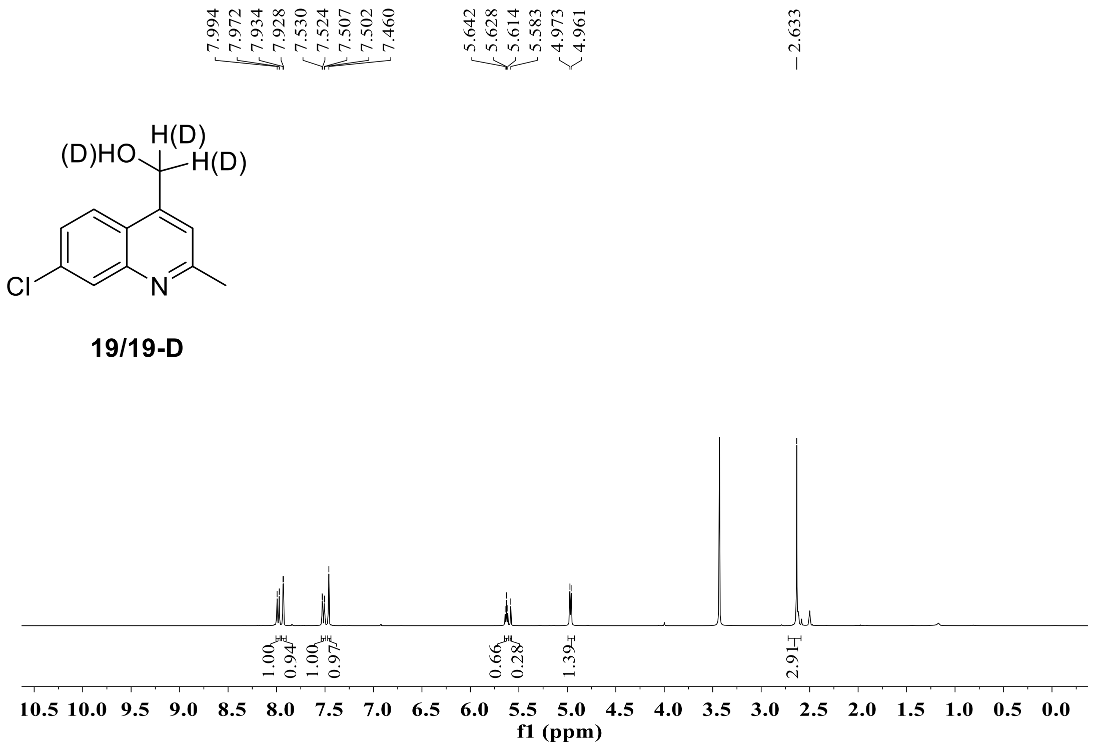

Supplementary Figure 41. ^{1}H NMR spectrum of desired product from KIE experiment.

Supplementary Methods

General information: All manipulations were carried out by standard schlenk techniques. Unless otherwise stated, analytical grade solvents and commercially available reagents were used to conduct the reactions. LED irradiation was accomplished using the blue photochemical reactors. Thin layer chromatography (TLC) employed glass 0.25 mm silica gel plates. Flash chromatography columns were packed with 200-300 mesh silica gel. Gradient flash chromatography was conducted and eluted with a continuous gradient from petroleum ether to ethyl acetate. All new compounds were characterized by ^{1}H NMR, ^{13}C NMR and HRMS. The known compounds were characterized by ^{1}H NMR and ^{13}C NMR. ^{1}H NMR and ^{13}C NMR spectra were recorded on a Bruker 400 MHz NMR spectrometer. The chemical shifts (δ) were given in part per million relative to internal tetramethyl silane (TMS, 0 ppm for ^{1}H NMR), $CDCl_3$ (77.16 ppm for ^{13}C NMR) and DMSO-d_6 (2.50 ppm for ^{1}H NMR, 39.52 ppm for ^{113}C NMR), respectively. High resolution mass spectra (HRMS) were measured with a Bruker UltiMate3000 & Compact instrument and accurate masses were reported for the molecular ion + Hydrogen (M+H). EPR spectra were recorded on a Bruker X-band A200 spectrometer. GC-MS spectra were recorded on Varian GC MS 3900-2100T or SHIMADZU GC MS-2010.

General procedure A for heteroarene scope: A solution of heteroarene (0.3 mmol, 1.0 equiv), 1.5 mL ethanol or methanol, selectfluor (0.6 mmol, 2.0 equiv, 212.5 mg) and TFA (0.45 mmol, 1.5 equiv, 51.3 mg) in degassed dry CH_3CN (2.0 mL) were stirred under nitrogen atmosphere and irradiated by 3 W blue LEDs at 25 ˚C for 24 h. After completion of the reaction, the reaction system was quenched by saturated $NaHCO_3$ aqueous solution. The aqueous solution was extracted with ethyl acetate (3 × 10 mL) and the combined extracts were dried with anhydrous Na_2SO_4. The solvents were removed under reduced pressure by rotary evaporation. Then, the pure product was obtained by flash column chromatography on silica gel (eluent: petroleum ether/ethyl acetate).

General procedure B for alcohol scope: A solution of isoquinoline (0.3 mmol, 1.0 equiv, 38.7 mg) or 7-chloro-2-methylquinoline (0.3 mmol, 1.0 equiv, 53.3 mg), alcohol (6.0 mmol, 20 equiv), selectfluor (0.6 mmol, 2.0 equiv, 212.5 mg) and TFA (0.45 mmol, 1.5 equiv, 51.3 mg) in degassed dry CH_3CN (2.0 mL)（additional 0.75 mL DCE was added for **25**) were stirred under nitrogen atmosphere and irradiated by 3 W blue LEDs at 25 ˚C for 24 h. After completion of the reaction, the reaction system was quenched by saturated $NaHCO_3$ aqueous solution. The aqueous solution was extracted with ethyl acetate (3 × 10 mL) and the combined extracts were dried with anhydrous Na_2SO_4. The solvents were removed under reduced pressure by rotary evaporation. Then, the pure product was obtained by flash column chromatography on silica gel (eluent: petroleum ether/ethyl acetate).

Procedure for Gram-Scale Reaction: A solution of isoquinoline **12** (9.9 mmol, 1.0 equiv, 1.16 g), 20 mL ethanol, selectfluor (19.8 mmol, 2.0 equiv, 6.37 g) and TFA (13.5 mmol, 1.5 equiv, 1.54 g) in degassed dry CH_3CN (20 mL) were stirred under nitrogen atmosphere and irradiated by 3 W blue LEDs at 25 ˚C for 97 h. After completion of the reaction, the reaction system was quenched by saturated $NaHCO_3$ aqueous solution. The aqueous solution was extracted with ethyl acetate (3 × 30 mL) and the combined extracts were dried with anhydrous Na_2SO_4. The solvents were removed under reduced pressure by rotary evaporation. Then, the pure product was obtained by flash column chromatography on silica gel (eluent: petroleum ether/ethyl acetate= 5:1).

Procedure for EPR studies: A solution of selectfluor (0.6 mmol, 212.5 mg) in degassed dry CH_3CN (2.0 mL) were stirred under nitrogen atmosphere, which was irradiated by 3 W blue LEDs

or under darkness at 25 ˚C for 2 h. Then, DMPO (30 μL) was added into the reaction system, which was further irradiated by 3 W blue LEDs or under darkness at 25 ˚C for 10 mins. The solution sample was taken out into a small tube and analyzed by EPR. EPR spectra was recorded at room temperature on EPR spectrometer operated at 9.821452 GHz. Typical spectrometer parameters are shown as follows, scan range: 150 G; center field set: 3504.54 G; time constant: 163.84 ms; scan time: 30 s modulation amplitude: 1.0 G; modulation frequency: 100 kHz; receiver gain: 1.00×104; microwave power: 21.59.

Procedure for Intermolecular Competition Experiment: A solution of isoquinoline **12** (0.3 mmol, 1.0 equiv, 38.7 mg), 1.0 mL ethanol, 0.5 mL diethyl ether, selectfluor (0.6 mmol, 2.0 equiv, 212.5 mg) and TFA (0.45 mmol, 1.5 equiv, 51.3 mg) in degassed dry CH_3CN (2.0 mL) were stirred under nitrogen atmosphere and irradiated by 3 W blue LEDs at 25 ˚C for 24 h. Afterwards, the reaction system was quenched by saturated $NaHCO_3$ aqueous solution. The aqueous solution was extracted with ethyl acetate (3 × 10 mL) and the combined extracts were dried with anhydrous Na_2SO_4. The reaction results were monitored by TLC and GC-MS. The solvents were removed under reduced pressure by rotary evaporation. Only pure product **13** was obtained in 66% by flash column chromatography on silica gel.

Procedure for Radical Inhibition Experiments: A solution of isoquinoline **12** (0.3 mmol, 1.0 equiv, 38.7 mg), 1.5 mL ethanol, selectfluor (0.6 mmol, 2.0 equiv, 212.5 mg), TFA (0.45 mmol, 1.5 equiv, 51.3 mg) and TEMPO (2,2,6,6-Tetramethyl-1-piperidinyloxy, 0.3 mmol, 1.0 equiv, 46.9 mg) in degassed dry CH_3CN (2.0 mL) were stirred under nitrogen atmosphere and irradiated by 3 W blue LEDs at 25 ˚C for 24 h. Afterwards, the reaction system was quenched by saturated $NaHCO_3$ aqueous solution. The aqueous solution was extracted with ethyl acetate (3 × 10 mL) and the combined extracts were dried with anhydrous Na_2SO_4. The reaction results were monitored by TLC and GC-MS.

Procedure for Kinetic Isotope Effect (KIE) Experiment: A solution of 7-chloro-2-methylquinoline **33** (0.3 mmol, 1.0 equiv, 53.3 mg), 0.75 mL MeOH and 0.75 mL MeOD-d_3, selectfluor (0.6 mmol, 2.0 equiv, 212.5 mg) and TFA (0.45 mmol, 1.5 equiv, 51.3 mg) in degassed

dry CH_3CN (2.0 mL) were stirred under nitrogen atmosphere and irradiated by 3 W blue LEDs at 25 ˚C for 8 h. Afterwards, the reaction system was quenched by saturated $NaHCO_3$ aqueous solution. The aqueous solution was extracted with ethyl acetate (3 × 10 mL) and the combined extracts were dried with anhydrous Na_2SO_4. The solvents were removed under reduced pressure by rotary evaporation. Then, the product was obtained by flash column chromatography on silica gel (eluent: petroleum ether/ethyl acetate= 5:1) to afford 18% combined products. The ratio was K_H: K_D = 2.2 which was determined by ^{1}H NMR spectrum (See Supplementary Fig. 13 and Supplementary Fig. 41).

Analytical Data of Compounds

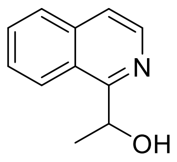

13

1-(Isoquinolin-1-yl)ethan-1-ol (13): 45.2 mg pale yellow liquid was obtained in 87% isolated yield. **^{1}H NMR** (400 MHz, $CDCl_3$) δ 8.44 (d, *J* = 5.6 Hz, 1H), 8.04 (d, *J* = 8.4 Hz, 1H), 7.86 (d, *J* = 8.2 Hz, 1H), 7.70 (t, *J* = 7.5 Hz, 1H), 7.65 – 7.56 (m, 2H), 5.59 (q, *J* = 6.5 Hz, 1H), 5.34 (s, 1H), 1.60 (d, *J* = 6.5 Hz, 3H). **^{13}C NMR** (101 MHz, $CDCl_3$) δ 162.21, 140.49, 136.52, 130.34, 127.62, 127.42, 124.65, 124.26, 120.58, 66.06, 25.50. **HRMS (ESI)** calcd for $C_{11}H_{12}NO^+$, $[M+H]^+$, 174.0913, found 174.0914.

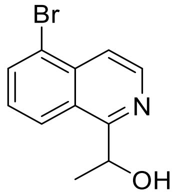

14

1-(5-Bromoisoquinolin-1-yl)ethan-1-ol (14): 43.8 mg pale yellow liquid was obtained in 58% isolated yield. **^{1}H NMR** (400 MHz, $CDCl_3$) δ 8.55 (d, *J* = 5.9 Hz, 1H), 8.07 – 7.87 (m, 3H), 7.58 – 7.41 (m, 1H), 5.59 (q, *J* = 6.2 Hz, 1H), 5.22 (s, 1H), 1.58 (d, *J* = 6.5 Hz, 3H). **^{13}C NMR** (101 MHz, $CDCl_3$) δ 162.71, 141.92, 135.65, 134.10, 127.74, 125.76, 123.91, 122.66, 119.50, 66.17, 25.58. **HRMS (ESI)** calcd for $C_{11}H_{11}BrNO^+$, $[M+H]^+$, 252.0019, found 252.0019.

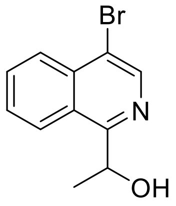

15

11-(4-Bromoisoquinolin-1-yl)ethan-1-ol (15): 49.7 mg pale yellow liquid was obtained in 66% isolated yield. **^{1}H NMR** (400 MHz, $CDCl_3$) δ 8.64 (s, 1H), 8.21 (d, *J* = 8.5 Hz, 1H), 8.04 (d, *J* = 8.5 Hz, 1H), 7.82 (ddd, *J* = 8.3, 7.0, 1.0 Hz, 1H), 7.69 (ddd, *J* = 8.2, 7.0, 1.1 Hz, 1H), 5.57 (t, *J* = 5.9 Hz, 1H), 4.99 (s, 1H), 1.59 (d, *J* = 6.5 Hz, 3H). **^{13}C NMR** (101 MHz, $CDCl_3$) δ 161.90, 142.30, 135.06, 131.62, 128.37, 127.01, 125.81, 124.58, 119.02, 66.10, 25.50. **HRMS (ESI)** calcd for $C_{11}H_{11}BrNO^+$, $[M+H]^+$, 252.0019, found 252.0020.

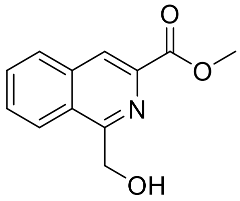

16

Methyl 1-(hydroxymethyl)isoquinoline-3-carboxylate (16): 29.9 mg pale yellow solid was obtained in 46% isolated yield. **^{1}H NMR** (400 MHz, DMSO-d_6) δ 8.57 (s, 1H), 8.44 (d, *J* = 7.9 Hz, 1H), 8.20 (d, *J* = 7.7 Hz, 1H), 7.94 – 7.78 (m, 2H), 5.59 (t, *J* = 5.7 Hz, 1H), 5.06 (d, *J* = 5.7 Hz, 2H), 3.93 (s, 3H). **^{13}C NMR** (101 MHz, DMSO-d_6) δ 165.66, 160.44, 139.42, 135.60, 131.08, 129.80, 128.75, 127.56, 126.03, 123.75, 63.84, 52.35. **HRMS (ESI)** calcd for $C_{12}H_{12}NO_3^+$, $[M+H]^+$, 218.0812, found 218.0822.

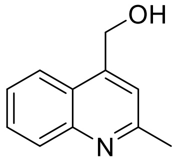

17

(2-Methylquinolin-4-yl)methanol (17): 40.5 mg white solid was obtained in 78% isolated yield. **^{1}H NMR** (400 MHz, DMSO-d_6) δ 7.95 (dd, *J* = 16.3, 8.1 Hz, 2H), 7.69 (ddd, *J* = 8.3, 6.9, 1.3 Hz, 1H), 7.52 (ddd, *J* = 8.1, 7.0, 1.1 Hz, 1H), 7.47 (s, 1H), 5.60 (s, 1H), 5.00 (s, 2H), 2.65 (s, 3H). **^{13}C NMR** (101 MHz, DMSO-d_6) δ 158.52, 147.78, 146.96, 129.09, 128.63, 125.52, 123.88, 123.31, 118.69, 59.69, 24.99. **HRMS (ESI)** calcd for $C_{11}H_{12}NO^+$, $[M+H]^+$, 174.0913, found 174.0912.

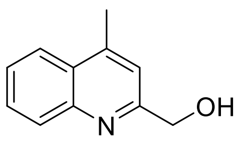

18

(4-Methylquinolin-2-yl)methanol (18): 27.5 mg white solid was obtained in 53% isolated yield. **^{1}H NMR** (400 MHz, DMSO-d_6) δ 8.09 – 8.01 (m, 1H), 7.96 – 7.90 (m, 1H), 7.72 (ddd, *J* = 8.3, 6.9, 1.4 Hz, 1H), 7.57 (ddd, *J* = 8.2, 6.9, 1.3 Hz, 1H), 7.51 (s, 1H), 5.55 (t, *J* = 5.9 Hz, 1H), 4.69 (d, *J* = 5.9 Hz, 2H), 2.68 (d, *J* = 0.8 Hz, 3H). **^{13}C NMR** (101 MHz, DMSO-d_6) δ 162.00, 146.70, 144.45, 129.27, 128.94, 126.90, 125.83, 124.18, 119.50, 64.82, 18.43. **HRMS (ESI)** calcd for $C_{11}H_{12}NO^+$, $[M+H]^+$, 174.0913, found 174.0914.

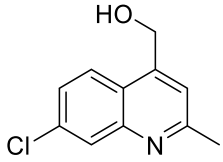

19

(7-Chloro-2-methylquinolin-4-yl)methanol (19): 46.6 mg white solid was obtained in 75% isolated yield. **^{1}H NMR** (400 MHz, DMSO-d_6) δ 8.00 (d, *J* = 8.9 Hz, 1H), 7.94 (d, *J* = 2.2 Hz, 1H), 7.53 (dd, *J* = 8.9, 2.2 Hz, 1H), 7.47 (s, 1H), 5.63 (t, *J* = 5.5 Hz, 1H), 4.97 (dd, *J* = 5.5, 0.9 Hz, 2H), 2.64 (s, 3H). **^{13}C NMR** (101 MHz, DMSO-d_6) δ 160.30, 148.01, 147.74, 133.64, 127.39, 127.35, 126.01, 125.62, 122.62, 119.30, 59.70, 25.09. **HRMS (ESI)** calcd for $C_{11}H_{11}ClNO^+$, $[M+H]^+$, 208.0524, found 208.0523.

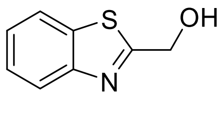

20

Benzo[d]thiazol-2-ylmethanol (20): 22.3 mg white solid was obtained in 45% isolated yield. **^{1}H NMR** (400 MHz, DMSO-d_6) δ 8.08 (d, *J* = 7.9 Hz, 1H), 7.92 (d, *J* = 8.1 Hz, 1H), 7.52 – 7.45 (m, 1H), 7.43 – 7.37 (m, 1H), 6.28 (t, *J* = 5.9 Hz, 1H), 4.87 (d, *J* = 6.0 Hz, 2H). **^{13}C NMR** (101 MHz, DMSO-d_6) δ 175.65, 153.09, 134.21, 126.01, 124.69, 122.32, 122.27, 61.29. **HRMS (ESI)** calcd for $C_8H_8NOS^+$, $[M+H]^+$, 166.0321, found 166.0325.

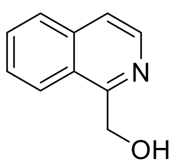

21

Isoquinolin-1-ylmethanol (21)[1]: 34.9 mg white solid was obtained in 73% isolated yield. **^{1}H NMR** (400 MHz, $CDCl_3$) δ 8.44 (d, *J* = 5.8 Hz, 1H), 7.95 – 7.83 (m, 2H), 7.71 (ddd, *J* = 8.2, 7.0, 1.1 Hz, 1H), 7.66 – 7.57 (m, 2H), 5.24 (s, 2H), 4.57 (s, 1H). **^{13}C NMR** (101 MHz, $CDCl_3$) δ 157.50, 140.47,

135.99, 130.63, 127.70, 127.50, 125.06, 123.30, 120.47, 61.52. **HRMS (ESI)** calcd for $C_{10}H_{10}NO^+$, $[M+H]^+$, 160.0757, found 160.0758.

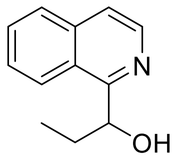

22

1-(Isoquinolin-1-yl)propan-1-ol (22): 41.0 mg pale yellow liquid was obtained in 73% isolated yield. **^{1}H NMR** (400 MHz, $CDCl_3$) δ 8.44 (d, *J* = 5.7 Hz, 1H), 8.07 – 7.99 (m, 1H), 7.86 (d, *J* = 8.2 Hz, 1H), 7.70 (ddd, *J* = 8.2, 6.9, 1.2 Hz, 1H), 7.65 – 7.56 (m, 2H), 5.49 – 5.36 (m, 1H), 5.18 (s, 1H), 2.17 – 2.00 (m, 1H), 1.71 (dp, *J* = 14.6, 7.3 Hz, 1H), 1.01 (t, *J* = 7.4 Hz, 3H). **^{13}C NMR** (101 MHz, $CDCl_3$) δ 161.27, 140.39, 136.48, 130.32, 127.61, 127.36, 124.92, 124.25, 120.51, 70.74, 32.08, 9.83. **HRMS (ESI)** calcd for $C_{12}H_{14}NO^+$, $[M+H]^+$, 188.1070, found 188.1070.

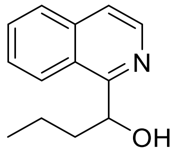

23

1-(Isoquinolin-1-yl)butan-1-ol (23): 47.8 mg pale yellow liquid was obtained in 79% isolated yield. **^{1}H NMR** (400 MHz, $CDCl_3$) δ 8.44 (d, *J* = 5.7 Hz, 1H), 8.04 (d, *J* = 8.4 Hz, 1H), 7.86 (d, *J* = 8.2 Hz, 1H), 7.75 – 7.67 (m, 1H), 7.66 – 7.55 (m, 2H), 5.48 (d, *J* = 4.4 Hz, 1H), 5.15 (s, 1H), 2.01 – 1.87 (m, 1H), 1.75 – 1.57 (m, 2H), 1.55 – 1.43 (m, 1H), 0.96 (t, *J* = 7.2 Hz, 3H). **^{13}C NMR** (101 MHz, $CDCl_3$) δ 161.62, 140.46, 136.50, 130.33, 127.63, 127.39, 124.85, 124.23, 120.50, 69.54, 41.55, 18.97, 14.15. **HRMS (ESI)** calcd for $C_{13}H_{16}NO^+$, $[M+H]^+$, 202.1226, found 202.1228.

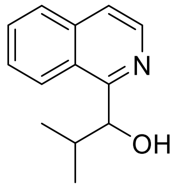

24

1-(Isoquinolin-1-yl)-2-methylpropan-1-ol (24): 40.4 mg pale yellow liquid was obtained in 67% isolated yield. **^{1}H NMR** (400 MHz, $CDCl_3$) δ 8.45 (d, *J* = 5.7 Hz, 1H), 8.04 (d, *J* = 8.4 Hz, 1H), 7.86 (d, *J* = 8.2 Hz, 1H), 7.70 (t, *J* = 7.5 Hz, 1H), 7.60 (dd, *J* = 14.7, 6.5 Hz, 2H), 5.36 (d, *J* = 2.0 Hz, 1H), 5.02 (s, 1H), 2.28 – 2.13 (m, 1H), 1.24 (d, *J* = 6.9 Hz, 3H), 0.62 (d, *J* = 6.7 Hz, 3H). **^{13}C NMR** (101 MHz, $CDCl_3$) δ 160.89, 140.25, 136.48, 130.27, 127.60, 127.27, 125.17, 124.39, 120.43, 73.76, 35.12, 20.85, 14.92. **HRMS (ESI)** calcd for $C_{13}H_{16}NO^+$, $[M+H]^+$, 202.1226, found 202.1226.

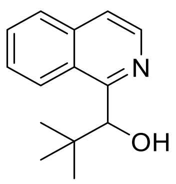

25

1-(Isoquinolin-1-yl)-2,2-dimethylpropan-1-ol (25): 23.9 mg white solid was obtained in 37% isolated yield. **^{1}H NMR** (400 MHz, DMSO-d_6) δ 8.78 (d, J = 8.6 Hz, 1H), 8.53 (d, J = 6.1 Hz, 1H), 8.19 (t, J = 7.9 Hz, 2H), 8.01 (t, J = 7.5 Hz, 1H), 7.85 (t, J = 7.7 Hz, 1H), 6.19 (s, 1H), 5.47 (s, 1H), 0.92 (s, 9H). **^{13}C NMR** (101 MHz, $CDCl_3$) δ 160.88, 140.69, 136.23, 130.06, 127.38, 126.94, 126.91, 125.62, 120.49, 75.55, 37.90, 26.76. **HRMS (ESI)** calcd for $C_{14}H_{18}NO^+$, $[M+H]^+$, 216.1383, found 216.1382.

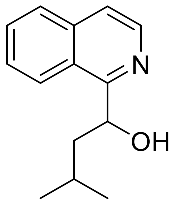

26

1-(Isoquinolin-1-yl)-2,2-dimethylpropan-1-ol (26): 49.0 mg pale yellow liquid was obtained in 76% isolated yield. **^{1}H NMR** (400 MHz, $CDCl_3$) δ 8.44 (d, J = 5.7 Hz, 1H), 8.01 (d, J = 8.4 Hz, 1H), 7.89 – 7.83 (m, 1H), 7.73 – 7.67 (m, 1H), 7.66 – 7.57 (m, 2H), 5.52 (d, J = 8.6 Hz, 1H), 5.07 (s, 1H), 2.27 – 2.10 (m, 1H), 1.66 (ddd, J = 13.8, 9.7, 2.4 Hz, 1H), 1.55 (ddd, J = 14.1, 10.2, 4.0 Hz, 1H), 1.20 (d, J = 6.6 Hz, 3H), 0.94 (d, J = 6.7 Hz, 3H). **^{13}C NMR** (101 MHz, $CDCl_3$) δ 162.09, 140.58, 136.55, 130.31, 127.67, 127.42, 124.74, 124.13, 120.49, 68.21, 49.00, 25.38, 24.01, 21.71. **HRMS (ESI)** calcd for $C_{14}H_{18}NO^+$, $[M+H]^+$, 216.1383, found 216.1384.

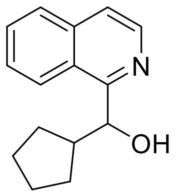

27

Cyclopentyl(isoquinolin-1-yl)methanol (27): 41.5 mg pale yellow liquid was obtained in 61% isolated yield. **^{1}H NMR** (400 MHz, $CDCl_3$) δ 8.44 (d, J = 5.7 Hz, 1H), 8.11 (d, J = 8.4 Hz, 1H), 7.86 (d, J = 8.2 Hz, 1H), 7.76 – 7.68 (m, 1H), 7.66 – 7.55 (m, 2H), 5.52 (d, J = 3.0 Hz, 1H), 5.02 (s, 1H), 2.43 (pd, J = 8.4, 3.6 Hz, 1H), 1.86 – 1.77 (m, 2H), 1.76 – 1.66 (m, 1H), 1.66 – 1.57 (m, 1H), 1.54 – 1.36 (m, 3H), 1.07 – 0.97 (m, 1H). **^{13}C NMR** (101 MHz, $CDCl_3$) δ 161.42, 140.45, 136.51, 130.30, 127.61, 127.30, 125.09, 124.45, 120.45, 71.16, 46.92, 30.01, 26.01, 25.86, 25.27. **HRMS (ESI)** calcd for $C_{15}H_{18}NO^+$, $[M+H]^+$, 228.1383, found 228.1382.

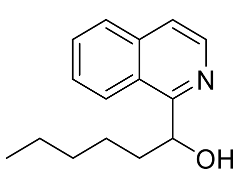

28

1-(Isoquinolin-1-yl)hexan-1-ol (28): 45.3 mg pale yellow liquid was obtained in 66% isolated yield. **^{1}H NMR** (400 MHz, $CDCl_3$) δ 8.44 (d, *J* = 5.7 Hz, 1H), 8.03 (d, *J* = 8.4 Hz, 1H), 7.85 (d, *J* = 8.2 Hz, 1H), 7.74 – 7.67 (m, 1H), 7.64 – 7.55 (m, 2H), 5.53 – 5.40 (m, 1H), 5.15 (s, 1H), 2.02 – 1.93 (m, 1H), 1.72 – 1.55 (m, 2H), 1.54 – 1.44 (m, 1H), 1.38 – 1.23 (m, 4H), 0.87 (t, *J* = 7.0 Hz, 3H). **^{13}C NMR** (101 MHz, $CDCl_3$) δ 161.65, 140.46, 136.50, 130.31, 127.62, 127.37, 124.86, 124.22, 120.47, 69.79, 39.44, 31.91, 25.47, 22.77, 14.18. **HRMS (ESI)** calcd for $C_{15}H_{20}NO^+$, $[M+H]^+$, 230.1539, found 230.1540.

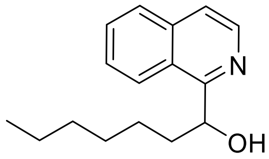

29

1-(Isoquinolin-1-yl)heptan-1-ol (29): 51.7 mg pale yellow liquid was obtained in 71% isolated yield. **^{1}H NMR** (400 MHz, $CDCl_3$) δ 8.44 (d, *J* = 5.7 Hz, 1H), 8.03 (d, *J* = 8.4 Hz, 1H), 7.86 (d, *J* = 8.2 Hz, 1H), 7.76 – 7.67 (m, 1H), 7.65 – 7.56 (m, 2H), 5.50 – 5.41 (m, 1H), 5.15 (s, 1H), 2.05 – 1.89 (m, 1H), 1.76 – 1.55 (m, 2H), 1.53 – 1.44 (m, 1H), 1.41 – 1.34 (m, 1H), 1.29 (d, *J* = 16.2 Hz, 6H), 0.86 (t, *J* = 6.8 Hz, 3H). **^{13}C NMR** (101 MHz, $CDCl_3$) δ 161.65, 140.46, 136.51, 130.32, 127.63, 127.38, 124.87, 124.23, 120.49, 69.79, 39.47, 31.94, 29.39, 25.74, 22.74, 14.22. **HRMS (ESI)** calcd for $C_{16}H_{22}NO^+$, $[M+H]^+$, 244.1696, found 244.1698.

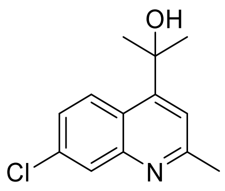

30

2-(7-Chloro-2-methylquinolin-4-yl)propan-2-ol (30): 17.6 mg pale yellow liquid was obtained in 25% isolated yield. **^{1}H NMR** (400 MHz, $CDCl_3$) δ 8.07 (d, *J* = 8.9 Hz, 1H), 7.91 (d, *J* = 2.0 Hz, 1H), 7.35 (dd, *J* = 8.9, 2.0 Hz, 1H), 6.59 (s, 1H), 4.92 – 4.68 (m, 1H), 2.67 (s, 3H), 1.47 (d, *J* = 6.1 Hz, 6H). **^{13}C NMR** (101 MHz, $CDCl_3$) δ 161.48, 160.67, 149.62, 135.63, 127.11, 125.46, 123.63, 118.97, 102.01, 70.85, 26.10, 21.85. **HRMS (ESI)** calcd for $C_{13}H_{15}ClNO^+$, $[M+H]^+$, 236.0837, found 236.0838.

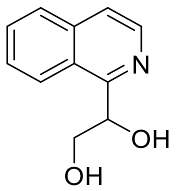

31

1-(Isoquinolin-1-yl)ethane-1,2-diol (31): 35.7 mg pale yellow solid was obtained in 63% isolated yield. **^{1}H NMR** (400 MHz, DMSO-d_6) δ 8.46 (t, *J* = 6.7 Hz, 2H), 7.97 (d, *J* = 8.1 Hz, 1H), 7.83 – 7.73 (m, 2H), 7.70 – 7.64 (m, 1H), 5.48 (d, *J* = 6.4 Hz, 1H), 5.34 (q, *J* = 6.1 Hz, 1H), 4.77 (t, *J* = 5.8 Hz, 1H), 3.91 – 3.73 (m, 2H). **^{13}C NMR** (101 MHz, DMSO-d_6) δ 160.42, 140.91, 136.03, 130.16, 127.22, 127.17, 126.05, 125.65, 120.35, 72.55, 65.87. **HRMS (ESI)** calcd for $C_{11}H_{12}NO_2^+$, $[M+H]^+$, 190.0863, found 190.0868.

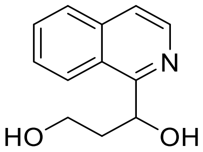

32

1-(Isoquinolin-1-yl)propane-1,3-diol (32): 41.4 mg pale yellow solid was obtained in 68% isolated yield. **^{1}H NMR** (400 MHz, DMSO-d_6) δ 8.48 – 8.36 (m, 2H), 7.98 (d, *J* = 8.1 Hz, 1H), 7.83 – 7.71 (m, 2H), 7.67 (ddd, *J* = 8.3, 6.9, 1.2 Hz, 1H), 5.51 – 5.43 (m, 1H), 5.38 (d, *J* = 6.5 Hz, 1H), 4.57 (t, *J* = 5.1 Hz, 1H), 3.71 – 3.60 (m, 1H), 3.59 – 3.50 (m, 1H), 2.10 – 2.00 (m, 1H), 1.99 – 1.90 (m, 1H). **^{13}C NMR** (101 MHz, DMSO-d_6) δ 162.23, 140.84, 136.13, 130.18, 127.36, 127.21, 125.34, 125.26, 120.24, 68.39, 57.94, 40.59. **HRMS (ESI)** calcd for $C_{12}H_{14}NO_2^+$, $[M+H]^+$, 204.1019, found 204.1022.

Supplementary References

1. Weitgenant, J. A., Mortison, J. D. & Helquist, P. Samarium-promoted coupling of pyridine-based heteroaryl analogues of benzylic acetates with carbonyl compounds, *Org. Lett.* **7**, 3609–3612 (2005).
